# Copper amine oxidases catalyze the oxidative deamination and hydrolysis of cyclic imines

**DOI:** 10.1038/s41467-018-08280-w

**Published:** 2019-01-24

**Authors:** Toshiki Nagakubo, Takuto Kumano, Takehiro Ohta, Yoshiteru Hashimoto, Michihiko Kobayashi

**Affiliations:** 1 Graduate School of Life and Environmental Sciences, University of Tsukuba, Ibaraki, Japan; 2 Graduate School of Life Science, University of Hyogo, Hyogo, Japan; 3 Present Address: Department of Applied Chemistry, Faculty of Engineering, Sanyo-Onoda City University, Yamaguchi, Japan

## Abstract

Although cyclic imines are present in various bioactive secondary metabolites, their degradative metabolism remains unknown. Here, we report that copper amine oxidases, which are important in metabolism of primary amines, catalyze a cyclic imine cleavage reaction. We isolate a microorganism (*Arthrobacter* sp. C-4A) which metabolizes a β-carboline alkaloid, harmaline. The harmaline-metabolizing enzyme (HarA) purified from strain C-4A is found to be copper amine oxidase and catalyze a ring-opening reaction of cyclic imine within harmaline, besides oxidative deamination of amines. Growth experiments on strain C-4A and Western blot analysis indicate that the HarA expression is induced by harmaline. We propose a reaction mechanism of the cyclic imine cleavage by HarA containing a post-translationally-synthesized cofactor, topaquinone. Together with the above results, the finding of the same activity of copper amine oxidase from *E. coli* suggests that, in many living organisms, these enzymes may play crucial roles in metabolism of ubiquitous cyclic imines.

## Abstract (web summary)

Little is known about the degradation pathway of cyclic imines that are frequently found in bioactive secondary metabolites. Here, the authors found and characterised a copper amine oxidase, HarA that catalyses a ring-opening reaction of cyclic imine in harmaline and oxidative deamination of amines.

## Introduction

β-Carboline alkaloids comprise a large group of indole alkaloids. They are widely distributed secondary metabolites in nature and exhibit remarkable bioactivities^1^. Some of them are candidate therapeutic agents for drug abuse^1^. In addition, β-carboline alkaloids such as reserpine (antihypertensive), yohimbine (α-receptor antagonist), eudistomin (antivirus, etc.), and mitragynine (opioid-receptor agonist) have been studied pharmacologically; in particular, the former two compounds have been used as medicines.

β-Carboline alkaloids are biosynthesized from tryptamine (or tryptophan) and an aldehyde through the Pictet–Spengler reaction. While this ring-closing reaction has been considered to be catalyzed by Pictet–Spenglerase family enzymes in nature, recent studies have shown that there is another enzyme family that synthesizes β-carboline alkaloids^2–5^. As endogenous compounds, possibly due to the wide distribution of these enzymes among organisms, β-carboline alkaloids are ubiquitously produced by broad species of plants, microorganisms and animals, including humans^6,7^. Although the biosynthesis of β-carboline alkaloids has been revealed, as described above, their degradative metabolism is unclear.

*Peganum harmala* is well-known as a medicinal plant, exhibiting cardiovascular, anti-depressant, antitumor and antibacterial effects^6^. Harmaline, one of the simplest β-carboline alkaloids, is responsible for these effects of *P. harmala*. Various parts of *P. harmala* including its seeds, fruit, roots and bark have been ingested as folk medicines for a long time in Middle Eastern, African and European countries^6^. Unique biological functions of harmaline, which is a bioactive compound in *P. harmala*, have been investigated, such as (i) an increasing effect on NO-release from endothelial cells, (ii) an inhibitory effect on monoamine oxidase A, and (iii) binding abilities with DNA, RNA and various receptors in the central nervous system^6^. While the worldwide use and such bioactivities of harmaline have attracted the interest of researchers for decades, to the best of our knowledge, degradation of harmaline has never been reported. As harmaline should exist in the soil in which a harmaline-producing plant, such as *P. harmala*, grows, we hypothesized that harmaline is degraded and metabolized by microorganisms in the soil.

Here, we describe the discovery of copper amine oxidase (CAO) as a harmaline-metabolic enzyme, from a harmaline-metabolizing microorganism. We also propose a two-step reaction mechanism for the cleavage of the C=N bond (in the unique cyclic imine structure), and show the generality of the dual functions of CAOs across species.

## Results

### Isolation and identification of harmaline-metabolizing bacteria

We collected the samples for the screening of harmaline-metabolizing microorganisms from the soil around the root of *P. harmala*, which was grown at the Tokyo Metropolitan Institute of Public Health. At ~2 weeks from the start of the screening, using the enrichment culture method described under Methods, we isolated five microorganisms that were able to grow on minimum media containing 0.025% (w/v) harmaline as the sole carbon source. From the isolated microorganisms, we selected one isolate, strain C-4A, that showed the highest harmaline-converting activity in the resting cell reaction, for further studies. Notably, strain C-4A was also able to metabolize harmaline as the sole nitrogen source.

Phylogenetic analysis revealed that the strain is closely related to the genus *Arthrobacter*, showing 99% 16S rRNA gene sequence similarity to an actinomycete, *Arthrobacter nitroguajacolicus* G2–1.

### Purification of the harmaline-metabolizing enzyme

**Fig. 1:**
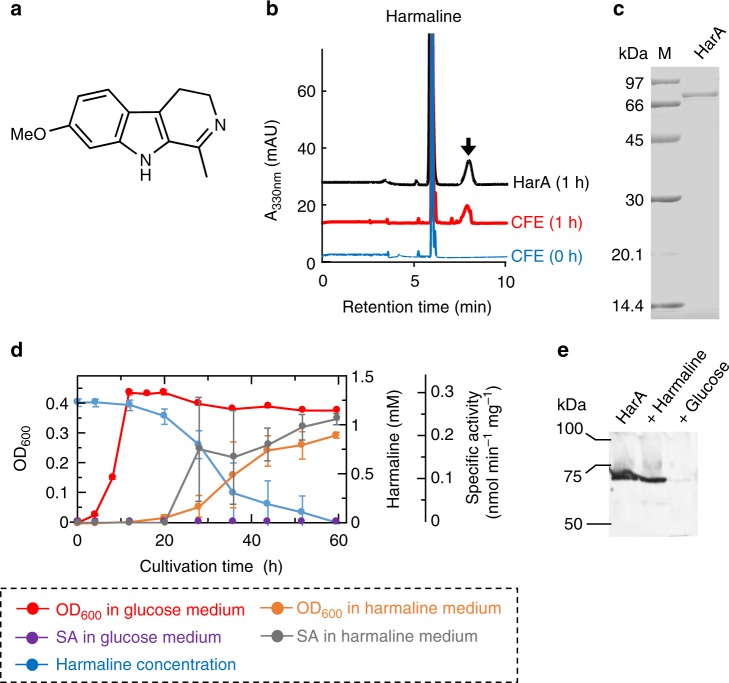
Discovery of a harmaline-metabolizing enzyme. Harmaline-metabolizing enzyme HarA was found from strain C-4A. **a** Structure of harmaline. **b** LC/MS analyses of the reaction product of harmaline. Reaction mixtures containing harmaline and each of a cell-free extract of strain C-4A and purified HarA were incubated and then analyzed at 330 nm. The arrow indicates the reaction product that exhibits *m/z* 230 [M−H]^−^ in the negative ion mode. **c** SDS-PAGE of purified HarA. Lane M, marker proteins: phosphorylase *b* (97 kDa), bovine serum albumin (66 kDa), ovalbumin (45 kDa), carbonic anhydrase (30 kDa), soybean trypsin inhibitor (20.1 kDa), and α-lactalbumin (14.4 kDa). The final concentration and purity of HarA were 0.186 mg ml^−1^ and ~95%, respectively. Source data are provided as a Source Data file. **d** Time courses of cell growth, harmaline concentration and specific activity (SA) for harmaline degradation in cell-free extracts during culture using media that contained harmaline or glucose as the sole carbon source. All the experiments were conducted in triplicate, and all data points represent the mean values ± S.D. for three experiments. Source Data are provided as a Source Data file. **e** Western blot analyses for purified HarA and cell-free extracts of C-4A grown in each of the media in **d**. The amount of purified HarA (left) was 20 ng. The amounts of cell-free extracts of C-4A grown in media containing harmaline (center) or glucose (right) as the sole carbon source were 15 μg. Source data are provided as a Source Data file

We incubated cell-free extracts of strain C-4A with harmaline (Fig. 1a) as a substrate. Liquid chromatography/mass spectrometry (LC/MS) analysis revealed that a reaction product exhibited *m/z* 230 [M−H]^−^ in the negative ion mode (Fig. 1b). Strain C-4A was cultured and harvested in 48 L of the above minimum medium, and a harmaline-metabolizing enzyme was then purified from the harvested cells by hydrophobic interaction and anion exchange chromatographies (Supplementary Table 1). The purified enzyme gave a single band corresponding to a molecular mass of 71 kDa on SDS-PAGE (Fig. 1c). The molecular mass of the native enzyme was shown to be 130 kDa on gel filtration chromatography, indicating that this enzyme consists of two identical subunits (Supplementary Fig. 1).

### Identification of the harmaline-metabolizing enzyme

We determined the draft genome sequence for strain C-4A using a next-generation sequencer. A local BLAST search was made for the draft genome sequence using a partial amino acid sequence (MHSPLFDSLTADEITMVSTL) of the purified enzyme. As a result, the gene coding for the harmaline-metabolizing enzyme (named HarA) was identified. The amino acid sequence of HarA showed identity (41%) with that of copper amine oxidase (CAO) of *Arthrobacter globiformis*^8^. Based on this finding, we examined whether or not HarA exhibited amine oxidase activity. On LC/MS analyses, we found that HarA catalyzed the oxidation of the amine moieties of tryptamine and serotonin (1 mM each).

In addition, HarA was heterologously expressed in *E. coli* and physicochemically characterized (Supplementary Figs. 2–5 and Supplementary Note 1). These analyses revealed that HarA contained Cu^2+^ and the coordinated metal ion would be essential for HarA activity.

### Time-dependent harmaline metabolism during cultivation

Strain C-4A was grown in the minimum medium containing each of glucose and harmaline as the sole carbon source (Fig. 1d). In the medium containing harmaline as the sole carbon source, strain C-4A exhibited full growth, degrading harmaline. Notably, no HarA activity was detected in the cell-free extract when C-4A was grown in the medium containing glucose as the sole carbon source, although the activity was observed in the cell-free extract cultured in the medium containing harmaline as the sole carbon source. On immunoblot analysis, in addition, HarA was detected in the cell-free extract of strain C-4A that was grown in the above harmaline-containing medium (Fig. 1e).

### Structure determination of the reaction product

**Fig. 2:**
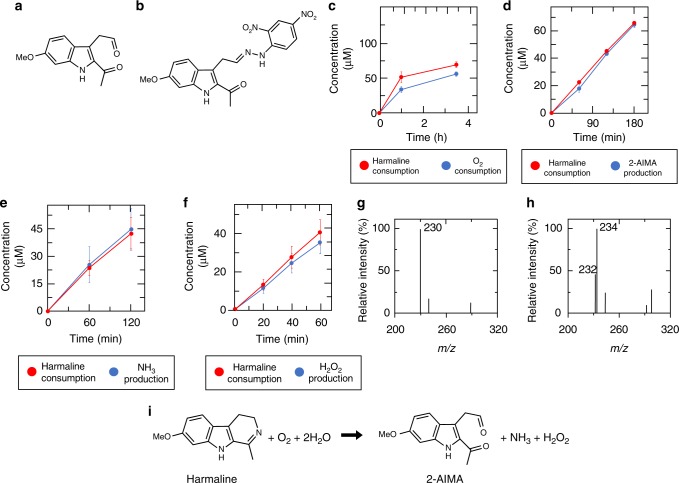
Identification of the reaction formula of HarA-catalyzed reaction. The reaction products and the stoichiometry of HarA-catalyzed harmaline degradation were determined. Structures of 2-AIMA and DNPH-derivatized 2-AIMA are shown in **a** and **b**, respectively. Each of consumption of **c** O_2_ and productions of **d** 2-AIMA, **e** NH_3_, and **f** H_2_O_2_ were measured. They are plotted in each panel together with the amounts of consumed harmaline during the reaction under the same conditions. All the assays were performed independently under the conditions in which reaction times and enzyme concentrations were optimized for each of the detection methods. All the assays were conducted in triplicate, and all data points represent the mean values ± S.D. for three experiments. Source data are provided as a Source Data file. MS spectra of 2-AIMA which were synthesized in 100% H_2_^16^O or 90% H_2_^18^O/10% H_2_^16^O are shown in **g** and **h**, respectively. **i** The reaction formula for HarA-catalyzed harmaline degradation. Two oxygen atoms of H_2_O_2_ are derived from O_2_

We next determined the structure of the reaction product that was yielded through HarA-catalyzed harmaline degradation and detected on LC/MS analysis (Fig. 1b). Structural analyses of the 2,4-dinitrophenylhydrazine (DNPH)-derivatized reaction product revealed that harmaline was converted into a compound, 2-acetyl-1*H*-indol-6-methoxy-3-acetaldehyde (2-AIMA), by HarA (Fig. 2a, b, Supplementary Figs. 6–11, Supplementary Table 2 and Supplementary Note 2). We then confirmed that HarA formed no by-product; during the HarA-catalyzed harmaline-degrading reaction, only the peak derived from 2-AIMA was observed in the LC chromatogram and MS spectrum of the corresponding compound (Supplementary Fig. 12).

Furthermore, 2-AIMA was found to be metabolized to a stable alcohol metabolite by strain C-4A (Supplementary Figs. 13–18, Supplementary Table 3 and Supplementary Note 3).

### Identification of other reaction products and stoichiometry

Identification of one of the reaction products as 2-AIMA, which is a indole derivative containing acetaldehyde and acetyl groups, indicated that the nitrogen atom of the cyclic imine within harmaline was removed through a HarA-catalyzed reaction. We thus investigated whether or not any nitrogen-containing compounds, such as HNO, NO, NO_2_^−^, NO_3_^−^, and NH_3_, could be detected during harmaline degradation. As a result, only NH_3_ was detected successfully (Supplementary Fig. 19). In addition, we found that H_2_O_2_ was also produced through HarA-catalyzed harmaline degradation. Also, HarA showed no activity under strict anaerobic conditions. These findings indicated that O_2_ acted as an electron acceptor and was converted into H_2_O_2_ in HarA-catalyzed harmaline degradation.

The stoichiometry of HarA-catalyzed harmaline degradation was examined. Consumption of O_2_ and formation of 2-AIMA, NH_3_ and H_2_O_2_, were stoichiometric with consumption of harmaline (Fig. 2c–f). In addition, we found that two oxygen atoms of 2-AIMA would be derived from H_2_O (Fig. 2g, h, Supplementary Figs. 20, 21 and Supplementary Note 4). Based on these findings, we determined HarA-catalyzed harmaline degradation as follows: C_13_H_14_N_2_O (harmaline) + O_2_ + 2H_2_O → C_13_H_13_NO_3_ (2-AIMA) + NH_3_ + H_2_O_2_ (Fig. 2i).

### Identification of topaquinone of HarA

**Fig. 3:**
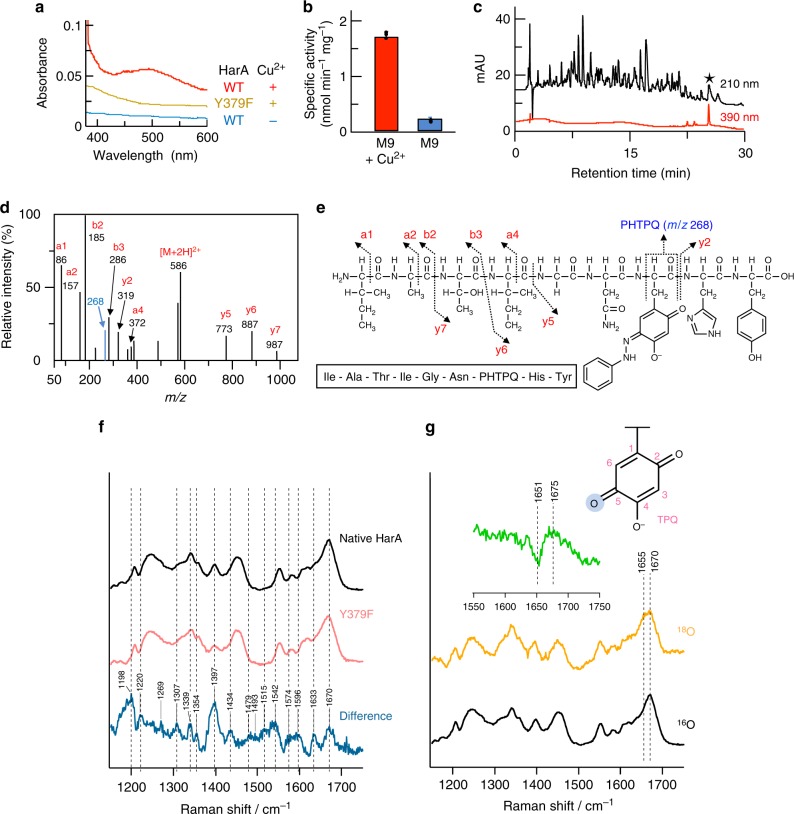
Post-translationally-synthesized cofactor topaquinone of HarA. Topaquinone (TPQ) within HarA was post-translationally synthesized from a specific tyrosine residue of HarA in the presence of copper ion and involved in HarA-catalyzed harmaline degradation. **a** Wild-type HarA and the Y379F mutant were expressed in *E. coli* in M9 medium with or without supplementation of 5 μM CuSO_4_. UV-vis spectra of the purified enzymes are shown. **b** Specific activities of wild-type HarA expressed in *E. coli* in M9 medium with or without supplementation of 5 μM CuSO_4_. All data points represent the mean values ± S.D. for three experiments. Source data are provided as a Source Data file. **c** Phenylhydrazine-treated HarA was digested with chymotrypsin, and then analyzed by LC/MS. The nonapeptide that was labeled with phenylhydrazine is denoted as a black star. **d** The above nonapeptide was then analyzed by LCMS/MS. **e** The fragment that exhibited a *m/z* value of 268 corresponds to that of a fragment containing the pheynylhydrazine-TPQ adduct (PHTPQ). **f** Raman spectra of native HarA and the Y379F mutant, and their difference (native HarA − Y379F). The concentration of each enzyme was 2 mM. **g** HarA was incubated in 94% H_2_^18^O for 2 days at 4 °C. Inset: Their difference (^16^O − ^18^O)

As described under Identification of the harmaline-metabolizing enzyme, HarA showed sequence similarity to CAO. CAOs contain a post-translationally-synthesized cofactor, topaquinone (TPQ), in their active sites. The UV-vis spectrum of HarA showed a peak, whose *λ*_max_ (489 nm, Fig. 3a) corresponded to that (470–500 nm) of TPQ in so far known CAOs. On the other hand, Cu-depleted HarA, which was heterologously expressed in M9 medium without the addition of Cu^2+^, showed extremely low levels of TPQ generation and harmaline-converting activity (Fig. 3a, b). TPQ was also detected as a phenylhydrazine adduct in the nonapeptide (Ile-Ala-Thr-Ile-Gly-Asn-TPQ-His-Tyr) that was derived from phenylhydrazine-treated HarA (Fig. 3c–e). The ratio of TPQ to HarA monomer was calculated by phenylhydrazine titration to be approximately 1:3 (Supplementary Fig. 22).

The previous study^9^ has revealed that the C5 oxygen of TPQ is specifically labeled when the CAO from *Arthrobacter globiformis* is exposed to H_2_^18^O. This ^18^O-substitution of the C5 oxygen has been demonstrated by Raman spectroscopic analyses, in which the frequency of the C5=O stretch shifted to lower energy through ^18^O-substitution. Using H_2_^18^O, we thus tried to identify the C5=O stretching band of TPQ in the Raman spectrum of HarA by means of the ^18^O-substitution. When HarA was exposed to H_2_^18^O, the C=O stretching band at 1670 cm^−1^ underwent a 24 cm^−1^ shift to lower energy with no shifts of other peaks (Fig. 3f, g). In addition, the frequency at 1670 cm^−1^ was similar to those of the C5=O stretches of TPQs of CAOs from *A. globiformis* (1683 cm^−1^), *E. coli* (1681 cm^−1^), and bovine serum (1678 cm^−1^)^9^. Thus, the oxygen isotope-sensitive band at 1670 cm^−1^ can be assigned as the C=O stretching mode of the C5 carbonyl of TPQ of HarA.

### Substrate specificity and kinetic properties of HarA

**Fig. 4:**
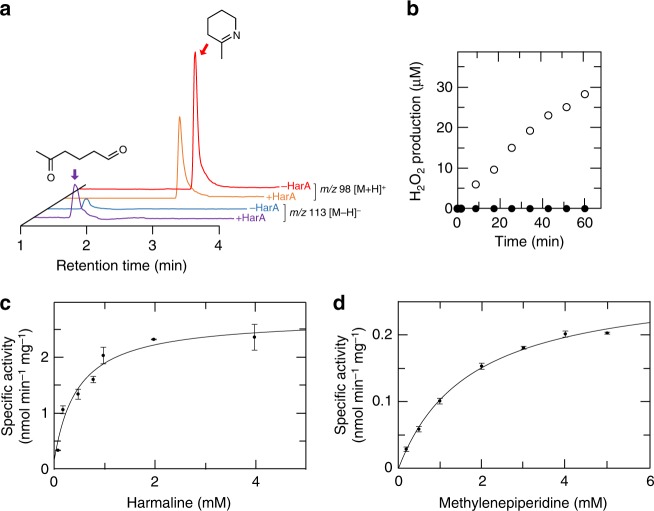
Substrate specificity and kinetic properties of HarA. HarA catalyzed ring-opening reaction of cyclic imine within 2-methylenepiperidine (MP). **a** After incubation with HarA, MP was converted into the reaction product (5-oxohexanal), which exhibited a *m/z* value of 113 [M−H]^−^. **b** Time-dependent H_2_O_2_ production during incubation of MP with or without HarA (shown in empty or filled circles, respectively) was measured by the DAOS method (described under Methods). Michaelis–Menten analyses were performed using **c** harmaline or **d** MP as the substrate. All data points in **c** and **d** represent the mean values ± S.E.M. for three experiments. Source data are provided as a Source Data file

We examined the substrate specificity of HarA using linear (*N*-benzylidenemethylamine and *N*-benzylideneaniline) or cyclic (2-methylenepiperidine [MP]) imine substrates (Supplementary Fig. 23). Among them, MP was found to be a substrate for HarA. The results of LC/MS and photometric analyses indicated that HarA cleaved the C-N bonds of MP to yield 5-oxohexanal and H_2_O_2_ (Fig. 4a, b). Linear imine substrates were inert for HarA.

Using various concentrations of harmaline and MP, we examined the kinetic properties of HarA. Typical hyperbolic curves of product formation for each of the harmaline and MP concentration were obtained, which indicated that the reactions followed Michaelis–Menten kinetics (Fig. 4c, d). The *K*_m_ value (1.70 mM) of HarA-catalyzed MP degradation was 3.87-fold higher than that (0.439 mM) of harmaline degradation. On the other hand, the *V*_max_ value (0.280 nmol min^−1^ mg^−1^) of HarA-catalyzed MP degradation was 9.89-fold lower than that (2.77 nmol min^−1^ mg^−1^) of harmaline degradation. These findings indicate that the aromatic ring moiety was important for the interaction between an imine substrate and HarA.

In addition, we also examined the substrate specificity of HarA toward amine substrates. HarA-catalyzed oxidations of aromatic substrates (tryptamine and benzylamine) were much faster than those of linear substrates (methylamine, ethylamine, hexylamine, and octylamine) (Supplementary Fig. 24).

### Construction and functional analyses of HarA mutants

The results of amino acid sequence alignment of HarA and CAO from *E. coli* (ECAO) indicated that four amino acid residues (Y281, D295, N378, and Y379) were conserved between ECAO and HarA, and located around the active site of HarA (Supplementary Fig. 25). D467, which would contribute to the stability of TPQ in ECAO^10^, was not conserved in HarA and corresponded to H380 of HarA in the sequence alignment.

**Fig. 5:**
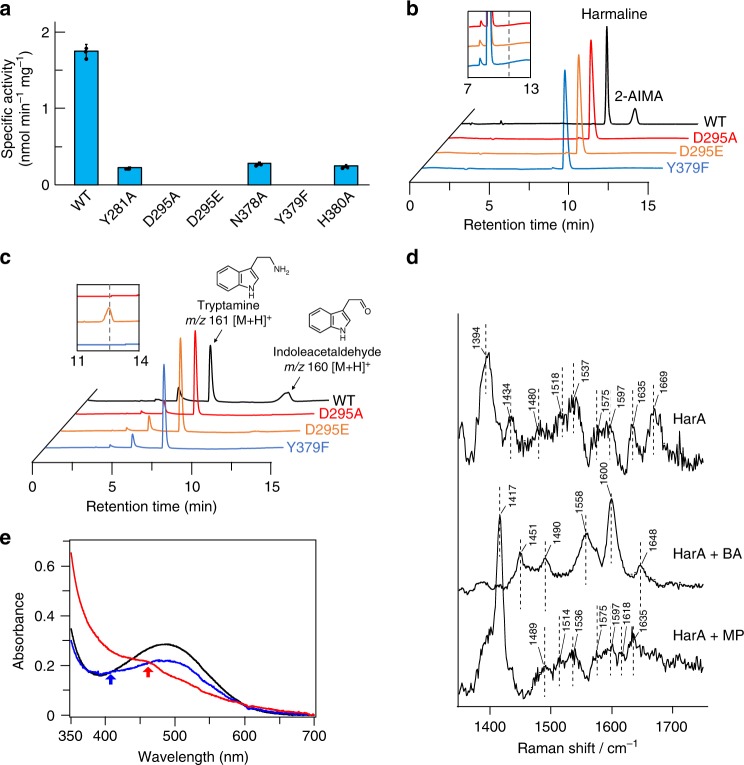
Identification of catalytic residues and reaction intermediates of HarA-catalyzed reaction. To obtain insight into the reaction mechanism of HatA-catalyzed cyclic imine cleavage reaction, various biochemical analyses were performed. **a** Specific activities of HarA mutants. All data points represent the mean values ± S.D. for three experiments. Source data are provided as a Source Data file. The reaction mixtures containing each of wild-type HarA, and the D295A, D295E and Y379F mutants were incubated with each of **b** harmaline and **c** tryptamine, and analyzed by LC/MS. Insets: magnified chromatograms (×10) of the three mutant enzymes. Dashed lines indicate the retention time in which each of the reaction products is detected. **d** Raman spectra of HarA without a substrate and with each of benzylamine (BA) and methylenepiperidine (MP) are shown. All spectra were obtained by subtracting the spectra of the Y379F mutant from those of native HarA. **e** UV-vis spectra of HarA were obtained with a spectrophotometer with an amine or imine substrate in an anaerobic environment. HarA was added to the reaction mixture containing 13 equivalents of benzylamine (blue), 5 equivalents of 2-methylenepiperidine (red), or 1% dimethylsulfoxide (black). Arrows indicate the peaks that appeared upon the addition of each substrate

Through single-amino acid substitution of each of the above amino acid residues, we constructed a set of mutants (i.e., Y281A, D295A, D295E, N378A, Y379F, and H380A, Supplementary Fig. 26). Among these mutants, the D295A, D295E, and Y379F ones were found to be completely inactive as to harmaline degradation (Fig. 5a, b). Even after overnight incubation, these mutants produced no reaction product. In addition, the D295A and Y379F mutations completely abolished the amine oxidase activity of HarA (Fig. 5c). On the other hand, D295E mutant catalyzed deamination of tryptamine, although the activity was less than that of wild-type enzyme (Fig. 5c). The results of spectroscopic analysis, and amino acid sequence alignment of HarA and ECAO indicated that Y379 is a precursor of TPQ (Supplementary Fig. 25, Fig. 3a). Considering the previous results^10,11^, the other tested amino acid residues would contribute to the stability of TPQ and the substrate specificity of the enzyme.

### Spectroscopic analyses of reaction intermediates

We spectroscopically analyzed reaction intermediates of harmaline degradation and oxidation of the amine moiety of an amine substrate. For Raman and UV-vis spectroscopic analyses, we were not able to use harmaline as a substrate for HarA for the following reasons: (i) the fluorescence of harmaline would have interfered with Raman spectroscopic analysis, and (ii) an absorbance band of harmaline in the UV-vis spectrum overlapped with that of TPQ. We thus used MP, which is not fluorescent and exhibits no absorbance above 300 nm in a UV-vis spectrum, as an imine substrate instead of harmaline in the following experiments.

For a clear picture of the resonance-enhanced modes of the TPQ cofactor, difference Raman spectra for HarA were obtained by subtracting the Y379F mutant spectra from that of native HarA (Supplementary Fig. 27). In the Raman spectrum of HarA with benzylamine, the C5=O stretching band at 1670 cm^−1^ disappeared, whereas some peaks appeared (Fig. 5d). These spectroscopic features were almost identical with those of a deprotonated imine intermediate of TPQ, which had been previously reported as the reaction intermediate of CAOs with amine substrates^9^. Notably, a newly appeared resonance-enhanced peak at 1600 cm^−1^ is indicative of the formation of an imine, which shows a C=N stretching band at the similar energy region^9^. When MP was used as a substrate, again the C5=O stretching band at 1669 cm^−1^ disappeared, while the overall spectral feature is quite different from an imine intermediate formed in the reaction with benzylamine (Fig. 5d). In addition, when HarA reacted with MP, *λ*_max_ of TPQ in the UV-vis spectrum shifted to around 460 nm from 489 nm (Fig. 5e). These spectroscopic features strongly suggested the existence of a reaction intermediate other than an imine intermediate of TPQ, when the substrate was a cyclic imine.

### Harmaline-degrading activity of CAO from *E. coli*

**Fig. 6:**
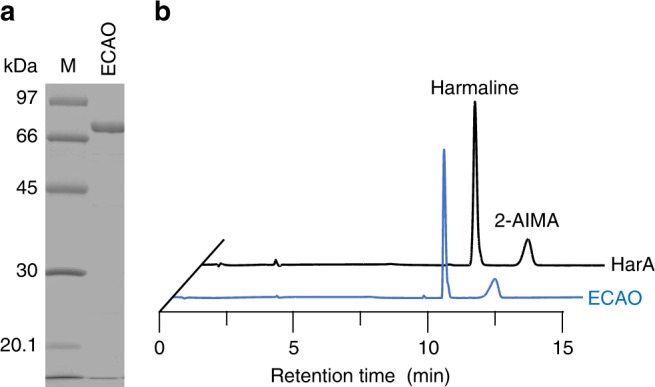
Harmaline-degrading activity of copper amine oxidase from *E. coli*. Copper amine oxidase (CAO) from *E. coli* (ECAO) was overexpressed in *E. coli* and purified. **a** SDS-PAGE of purified ECAO. Source data are provided as a Source Data file. **b** Harmaline-converting activity of ECAO. The concentrations of HarA and ECAO were 0.5 mg mL^−1^. Lane M, marker proteins

We investigated whether or not another CAO could catalyze harmaline degradation. The CAO-coding gene (*tynA*)^12^ was amplified from the genome of *E. coli* DH10B. An expression plasmid containing *tynA* was then constructed and introduced into *E. coli* DH10B. CAO from *E. coli* (ECAO) was expressed in *E. coli* BL21-CodonPlus(DE3)-RIL and purified (Fig. 6a). The purified ECAO was able to convert harmaline into 2-AIMA (Fig. 6b). However, the harmaline-degrading activity of *E. coli* DH10B was not induced on the addition of harmaline to the culture medium.

## Discussion

β-Carboline alkaloids have attracted interest due to their remarkable bioactivities. For example, mitragynine, which is present in kratom (*Mitragyna speciosa*) leaves, is one of the β-carboline alkaloids and has been investigated as a substitute for opium or as a cure for opium addiction^13^. Harmaline is also a β-carboline alkaloid and produced by many living organisms; it exists in humans^14^. One of the organisms is *Banisteriopsis caapi* (*Malpigbiaceae*), from which Amazonian tribes make a botanical beverage, Ayahuasca, for sacred rituals. Ayahuasca is famous for its strong hallucinogenic activity, and has the potential for medical use as a cure for drug addiction, depression, and anxiety disorders^15^. Notably, unique functions of harmaline, such as inhibition of monoamine oxidase A, are considered to contribute to these bioactivities. In addition, harmaline and harmine, which is a dehydrogenated derivative of harmaline, have been shown to exhibit potential therapeutic activity against diabetes^16^. Drug addiction, psychiatric diseases and diabetes are social problems in many countries, and β-carboline alkaloids may be keys for coping with such problems. On the other hand, the knowledge of their metabolism is limited. In mammals, only *O*-demethylation, hydroxylation, and dehydrogenation are known as metabolic reactions for harmaline^17,18^.

In nature, imines are generated through numerous metabolic pathways, such as biosynthetic pathways for secondary metabolites^19^. In addition, cyclic imines are present in the substructures of various bioactive secondary metabolites; e.g., gymnodimine (antagonist for nicotinic acetylcholine receptors)^20,21^, koranimine (antibiotic)^22^, and venoms of fire ants (inhibitors of nitric oxide synthase)^23^. Synthetic cyclic imine sugars as potent inhibitors of various glycosidases are of medicinal importance^24^. Notably, in contrast to linear imines, cyclic imines are generally stable and hardly undergo hydrolysis. In fact, cyclic imine within harmaline was found to be stable with the respect to the spontaneous hydrolysis in water (Supplementary Fig. 28 and Supplementary Note 5). As far as we know, cyclic imine-opening enzymes have never been previously found; cleaving activity of CAO toward cyclic imines including harmaline has never been reported. Although flavin-dependent monoamine oxidases (MAOs) catalyze similar oxidative deamination to that of CAOs, notably, harmaline and its derivatives have been known to be a strong competitive inhibitor of MAOs rather than their substrate^25^. Therefore, harmaline-degrading activity would be a unique feature of CAOs.

CAOs contain TPQ, which is considered to be generated via self-oxidation of the specific tyrosine residue by coordinated Cu^2+^ in their active sites^26,27^. Similar to TPQ, some cofactors, such as lysine tyrosylquinone (LTQ) and tryptophan tryptophylquinone (TTQ), are also known to be post-translationally generated from specific amino acid residues^28^. Among them, TPQ is the most ubiquitous, because TPQ-containing CAOs are widely distributed in bacteria, yeast, fungi, plants, and animals. This finding indicates that these CAOs are also able to catalyze imine-cleaving reactions if imine substrates bind to the active sites of the enzymes. Indeed, CAO from *E. coli* (ECAO), which shares similar substrate specificity for amine-oxidizing activity toward aromatic substrates^29^ with HarA, also exhibited harmaline-metabolizing activity (Fig. 6). In addition, human CAOs, such as AOC2 (retina-specific copper amine oxidase) and AOC3 (vascular adhesion protein, VAP-1), are known to have substrate specificities that are similar to those of HarA and ECAO; these enzymes are more specific for aromatic substrates, such as benzylamine or tryptamine, than methylamine or ethylamine^30,31^. Although overall amino acid sequences of ECAO and AOC3 exhibit low sequence identity (29%) with each other, their active site arrangements are similar to each other (Supplementary Fig. 25b)^12,32^. Moreover, notably, harmaline or its derivatives have been detected in human tissues such as the eyes of cataract patients, where AOC2 is specifically expressed^7,33^. Therefore, these observations indicate that β-carboline-metabolizing pathways involving CAOs may also be present in humans and other living organisms. The implication of the existence of the above metabolism in humans is important, because β-carboline alkaloids, including harmaline, have been shown to be endogenous compounds and to have therapeutic potency as to various diseases^6,15,33^.

Many studies have been performed to elucidate the mechanism of CAO-catalyzed amine oxidation. In the proposed mechanism^34–36^, TPQ forms substrate Schiff base with the substrate, an amine. Substrate Schiff base is converted into product Schiff base and then hydrolyzed by the aspartate residue in the active site of CAO, releasing an aldehyde product. Next, the copper ion activates O_2_ to accept electrons from the resulting semiquinone. Subsequently, NH_3_, H_2_O_2_, and TPQ are generated.

The results of single-amino acid mutagenesis showed that Asp295 of HarA is crucial for enzyme activity toward both harmaline and amine substrates. In addition, we concluded that coordination of the copper ion in the active site of HarA is essential for TPQ formation and HarA-catalyzed harmaline degradation, for the following reasons: (i) addition of Cu^2+^ to M9 medium was required for heterologous expression of HarA that contained the post-translationally-synthesized TPQ; (ii) chelating reagents (e.g., EDTA and diethyldithiocarbamate) decreased the activity of HarA; and (iii) the copper content of HarA was almost equivalent to that of TPQ. As the aspartate residue and copper ion have been shown to also be crucial for CAO-catalyzed amine oxidation^34–36^, the above results indicate that amine oxidation and harmaline-degrading reaction share these catalytic components (aspartate residue and copper ion).

**Fig. 7:**
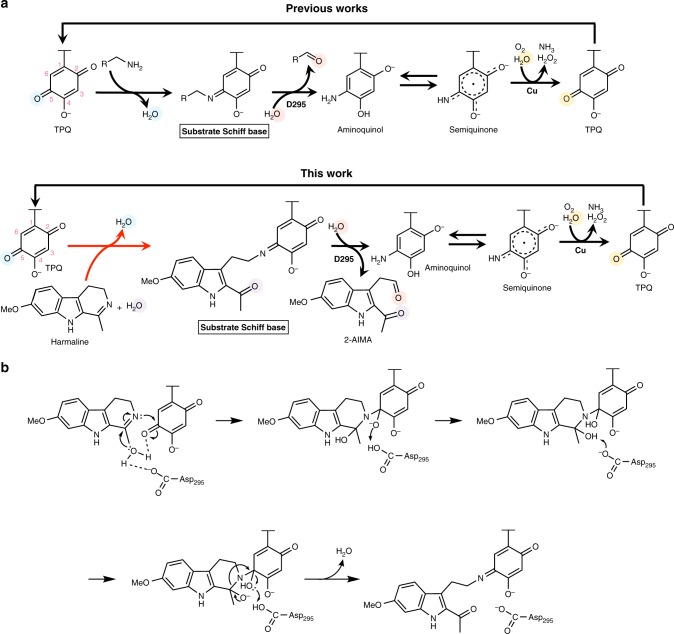
Schematic diagram of a possible reaction mechanism of HarA-catalyzed reactions. A possible reaction mechanism of two-step degradation of harmaline by HarA. **a** The reaction mechanisms of oxidation of an amine substrate (top) and degradation of harmaline (bottom) catalyzed by CAO. The reaction found in this study is indicated by a red arrow. Each substrate forms substrate Schiff base with TPQ. Substrate Schiff base is converted to product Schiff base (not shown in the figure) through deprotonation by deprotonated Asp295. Hydrolysis of product Schiff base is facilitated by protonation of the intermediate by protonated Asp295. **b** A possible reaction mechanism of the hydrolysis of cyclic imine and the formation of substrate Schiff base (indicated by the red arrow in **a**) when harmaline was used as the substrate

A semiquinone intermediate has been identified as one of the reaction intermediates (which are derived from TPQ) of CAO-catalyzed amine oxidation^9^. A previous study^9^ revealed that the semiquinone intermediate showed a weak resonance Raman effect and a peak at around 460 nm in the UV-vis spectrum. As these features are similar to those of a reaction intermediate of HarA with MP, it is indicated that this intermediate was also observed in our experiment. The existence of semiquinone in both HarA-catalyzed MP degradation and deamination of amine substrates suggests that these reactions proceed via the formation of so far known intermediates (i.e., substrate Schiff base, product Schiff base, aminoquinol and semiquinone) of CAO-catalyzed amine oxidation (Supplementary Fig. 29a). Based on the above findings, we propose that harmaline is degraded into 2-AIMA through a reaction mechanism that involves TPQ as a redox cofactor (Fig. 7a).

The previous study^9^ shows that some amine substrates form an inactive deprotonated imine intermediate with TPQ. Although substrate Schiff base and product Schiff base are unstable, the above inactive deprotonated imine intermediate is detectable on Raman spectroscopy. In our resonance Raman spectroscopic analyses, we observed a benzylamine adduct, which would be the inactive deprotonated imine intermediate, in HarA (Fig. 5d and Supplementary Fig. 27a). In the proposed reaction mechanism of CAOs^9^, nucleophilic attack on product Schiff base by H_2_O is facilitated by protonation of the intermediate. As reported previously for the methylamine adducts of phenylethylamine oxidase and bovine serum amine oxidase^9^, the deprotonated and inactive state of the benzylamine adduct of HarA may be favored by flipping of TPQ and interaction with copper or amino acid residues under anaerobic conditions (Supplementary Fig. 29b).

Here, HarA and ECAO were found to convert harmaline into 2-AIMA that included two oxygen atoms derived from H_2_O. One of these oxygen atoms would be incorporated into 2-AIMA through hydrolysis of product Schiff base. On the other hand, the other H_2_O-derived oxygen atom would be added to harmaline through nucleophilic attack by activated H_2_O on an electrophilic carbon before the formation of substrate Schiff base. The above result of oxygen incorporation suggests that H_2_O is activated in the initial step of the reaction by any of the active site components, which are polar amino acid residues, TPQ and copper. Considering the previous observations^37,38^, however, TPQ and copper are not likely to act as base catalysts for H_2_O activation in the imine-opening reaction for the following reasons: (i) the hydroxyl group at C4 of TPQ forms hydrogen bond with nearby tyrosine residue; and (ii) copper is distant from C5=O, with which an imine substrate would form product Schiff base (Supplementary Fig. 25b), of TPQ in the resting state. On the other hand, D295E mutant catalyzed the deamination of tryptamine, although this enzyme completely lacked harmaline-degrading activity (Fig. 5a–c). These results indicate that HarA lost the ability for the hydrolysis of harmaline through D295E mutation, while Glu295 could act as an active site base instead of Asp295 for oxidative deamination of an amine substrate, as previously reported for CAOs of *E. coli*^39^. Therefore, Asp295 may play an essential role for the hydrolysis of cyclic imine, and length of the side chain of the amino acid residue would be crucial for the activity. Based on our results and the previous observations^34–40^, we here propose a possible reaction mechanism in which H_2_O is activated by the active site component(s), such as Asp295, and the resultant activated H_2_O attacks on the electrophilic carbon of C=N within cyclic imine (Fig. 7b and Supplementary Fig. 29c); cyclic imine would be opened through the above hydrolytic reaction, and then form substrate Schiff base with TPQ. Therefore, CAOs would be unique dual functional enzymes catalyzing cyclic imine hydrolysis besides oxidative deamination of amine substrates. Future studies are necessary to clarify the detailed mechanism of harmaline degradation.

It is possible that the hydrolysis of cyclic imine within harmaline starts from the corresponding enamine. However, we consider that the hydrolysis of imine would be more likely than that of the enamine isomer in HarA-catalyzed harmaline degradation for the following reasons: (i) carbon atoms of C=C of the enamine are likely to be more electron-rich than the imine due to the presence of adjacent electron-donating nitrogen atom, and (ii) although protonation of C=C by an acid catalyst would be required for hydration of the enamine, the results of site-directed mutagenesis analyses of HarA show that there was no candidate amino acid residue which acts as the acid catalyst in HarA. Therefore, we concluded that the hydrolysis of the imine within harmaline would be the first step of HarA-catalyzed harmaline degradation.

The crystal structure of ECAO has revealed that one water molecule formed hydrogen bonds with aspartate residue and C5=O of TPQ in an active state of the enzyme^40^. In addition, we found that substitution of TPQ with Phe completely abolished harmaline-degrading activity of HarA (Fig. 5a, b). These observations and our results indicate that an active site environment which is created by TPQ and other amino acid residues would be a key for the ring-opening reaction of cyclic imines that are generally stable and hardly undergo hydrolysis. Involvement of TPQ may explain why harmaline was not degraded by imine reductases that catalyze hydrogenation of cyclic imine within harmaline although aspartate residue is considered to act as a proton donor in their active sites^41^.

In the present study, we show that a certain tyrosine residue of a harmaline-metabolizing enzyme, which is copper amine oxidase, is post-translationally modified in the presence of a copper ion and the resulting modified residue is involved in the harmaline-degrading reaction. Our findings would extend understanding of the catalytic abilities of ubiquitous and extensively studied CAOs. The dual functions of CAOs described here will lead to further elucidation of unidentified physiological roles of the enzymes.

## Methods

### Materials

Harmaline, tryptamine and serotonin were purchased from Tokyo Kasei Kogyo Co., Ltd. (Tokyo, Japan). *N*-Benzylidenemethylamine, *N*-benzylideneaniline, benzylamine, methylamine, and ethylamine were purchased from Wako Pure Chemical Industries, Ltd. (Osaka, Japan). 2-Methylenepiperidine was purchased from Enamine (Monmouth, NJ, USA). *N*-Ethyl-*N*-(2-hydroxy-3-sulfopropyl)-3,5-dimethoxyaniline was purchased from Dojindo (Kumamoto, Japan). All other chemicals used were from commercial sources and of analytical grade. Resource Q, HiPrep DEAE FF 16/10 and a low molecular weight standard kit were obtained from GE Healthcare (Buckinghamshire, UK).

### Isolation of harmaline-metabolizing microorganisms

Harmaline-metabolizing microorganisms were isolated from soil samples as follows. A spoonful of a soil sample around the roots of *Peganum harmala* was added to a test tube containing 10 ml of a screening medium (pH 7.0) consisting of 0.1% (w/v) (NH_4_)_2_SO_4_, 0.05% (w/v) K_2_HPO_4_, 0.05% (w/v) KH_2_PO_4_, 0.05% (w/v) MgSO_4_·7H_2_O, 0.0005% (w/v) FeSO_4_·7H_2_O, 0.025% (w/v) harmaline, and 20% (v/v) tap water. After incubation at 28 °C for 7 days, 1% (v/v) of the cultivated medium was added to the same fresh medium, followed by incubation at 28 °C for 7 days. This step was repeated two times. After enrichment, the culture broth was spread on agar plates, containing 1% (w/v) agar in the above screening medium, followed by incubation at 28 °C for 3 days. Colonies that grew on these plates were then isolated. Each of the isolated strains was inoculated into a test tube containing the above liquid screening medium, followed by incubation at 28 °C for 3 days. Cells were harvested by centrifugation (4000 × *g*, 10 min, 4 °C). After washing twice with 20 mM Hepes-NaOH (pH 7.0), cells were resuspended in the same buffer. Resuspended cells were then disrupted by sonication, and centrifuged (27,000 × *g*, 10 min, 4 °C) to prepare a cell-free extract.

### Enzyme assays

All of the reactions were performed under linear conditions with an appropriate amount of protein and a suitable reaction time, unless otherwise noted. Measurement of enzyme activity was performed as follows. One hundred microliters of the reaction mixture contained 0.05–1 mg mL^−1^ HarA, 20 mM Hepes-NaOH (pH 7.0), and 0.5–1 mM substrate (in DMSO). The reaction was initiated by adding the enzyme, followed by incubation at 28 °C. After incubation, the reaction was stopped by adding 100 μL of acetonitrile. The protein concentrations were determined with a protein assay kit (Nacalai Tesque Co., Inc., Kyoto, Japan) using bovine serum albumin as the standard, according to the method of Bradford^42^.

### Liquid chromatography and mass spectrometry

LC/MS analyses were carried out using a Prominence system with a photodiode array detector (SPD-M20A) and an LCMS-8030 (Shimadzu, Kyoto, Japan) equipped with a COSMOSIL πNAP column (4.6 × 150 mm; Nacalai Tesque Co., Inc.). The HPLC conditions were as follows: flow rate, 1 mL min^−1^; temperature, 40 °C; solvent A, 0.05% (v/v) formic acid; and solvent B, acetonitrile. After column equilibration with 100% solution A, 10 μl of a sample was injected into the column. The sample were eluted from the column by increasing the concentration of solvent B from 0 to 100% over 12 min.

### Purification of the harmaline-metabolizing enzyme

All purification procedures were performed at 0–4 °C. Cell-free extracts containing HarA were prepared as described above and fractionated with ammonium sulfate (35–80% saturation). The fractionated solutions were then applied to a Hiprep Butyl FF 16/10 column (20 mL) equilibrated with 20 mM Tris-HCl buffer containing 1 M ammonium sulfate (pH 7.5 at 4 °C). Protein was eluted from the column by decreasing the concentration of ammonium sulfate linearly from 1 to 0 M in the same buffer. The active fractions were collected and then dialyzed against 10 mM Tris-HCl buffer (pH 8.0 at 4 °C). The protein solution was applied to a Resource Q column (6 mL) equilibrated with 20 mM Tris-HCl (pH 8.0 at 4 °C). The protein was eluted by increasing the concentration of NaCl linearly from 0 to 400 mM in the same buffer. The active fractions were collected and then dialyzed as described above. The protein solution was applied to a BioAssist Q column (Tosoh Co., Ltd., Tokyo, Japan) equilibrated with the buffer used for Resource Q. Protein was eluted from the column by increasing the concentration of NaCl linearly from 0 to 400 mM in the same buffer.

Cell-free extracts containing ECAO were applied to a Hiprep DEAE FF 16/10 column (20 mL) equilibrated with 20 mM Tris-HCl buffer (pH 8.0 at 4 °C). Protein was eluted from the column by increasing the NaCl concentration linearly from 0 to 1 M in the same buffer.

### Draft genome sequence of strain C-4A

Strain C-4A was cultured at 28 °C for 16 h in 100 mL of 2× YT media. Cells were harvested and then washed with saline-EDTA (pH 8.0) containing 0.15 M NaCl and 0.1 M EDTA. In total, 1 mg mL^−1^ of lysozyme and 0.0867 mg mL^−1^ of proteinase K were added to the resultant suspension (11 mL) and incubated at 37 °C for 1 h. After incubation, 2 mL of 10% SDS was added to the solution and incubated at 55 °C for 1 h. DNA was extracted by phenol/chloroform/isoamylalcohol (25/24/1; v/v/v). After isopropanol precipitation of the DNA, the sample was treated with RNase. The treated DNA was extracted by isopropanol precipitation again. An Illumina Hiseq platform (Illumina, Inc., San Diego, CA, USA) was used for draft genome sequencing of strain C-4A. MiGAP (http://www.migap.org) was used for annotation of the draft genome sequence.

### Cloning and heterologous expression of *harA* and *tynA*

Bacterial strains and plasmids used in the following experiments are listed in Supplementary Table 4. *harA* was amplified using the genome of strain C-4A and the listed primers (Supplementary Table 5). The amplified *harA* gene was integrated with pETDuet-1 vector by In-Fusion (Clontech Laboratories Inc.). The resulting plasmid named pETDuet-*harA* was transformed into *E. coli* BL21-CodonPlus(DE3)-RIL. The transformed *E. coli* was cultivated in 16 L of liquid M9 media containing 5 μM CuSO_4_, 50 μg mL^−1^ ampicilin and 34 μg mL^−1^ chloramphenicol at 37 °C to an OD_600_ of 0.3. Isopropyl-β-D-thiogalactoside (IPTG) was added to a final concentration of 0.1 mM. After IPTG addition to the media, *E. coli* was cultivated at 18 °C for 20 h. Cells were harvested and disrupted by sonication. The lysate was centrifuged at 27,000 × *g* at 4 °C for 20 min. HarA was purified from the resultant cell-free extract by the method described under Purification of the harmaline-metabolizing enzyme.

*tynA*, which is the gene coding for copper amine oxidase, was amplified using the genome of *E. coli* DH10B and the listed primers (Supplementary Table 5). The amplified *tynA* gene was integrated with pET24a(+) vector by In-Fusion. The resulting plasmid named pET24a(+)-*tynA* was transformed into *E. coli* BL21-CodonPlus(DE3)-RIL. The copper amine oxidase was expressed by the above method using kanamycin instead of ampicilin.

### Determination of the molecular mass of HarA

HarA was applied to a Superdex 200 10/300 GL column (GE Healthcare UK Ltd., Little Chalfont, UK) using an AKTA purifier (GE Healthcare). HarA was eluted from the column under the following conditions: flow rate, 0.2 mL min^−1^; and buffer, 20 mM potassium phosphate buffer (pH 7.0) containing 200 mM NaCl. Thyroglobulin (670 kDa), lactate dehydrogenase (142 kDa), enolase (67 kDa), and cytochrome *c* (12.4 kDa), were applied before and after sample injection as standard proteins. The molecular mass of HarA was calculated based on the standard curve obtained from the mobilities of the standard proteins.

### Effect of pH on HarA activity

For pH stability analyses, after HarA had been incubated at 20 °C for 1 h in 20 mM Britton–Robinson buffer (pH 3.0–11.0), an aliquot of each enzyme solution was taken and then assayed in the standard reaction mixture. HarA activity was measured by HPLC. For pH dependency analyses, the reaction mixture containing 80 mM Britton–Robinson buffer (pH 3.0–11.0) was incubated at 28 °C, and then analyzed by HPLC.

### Western blot analysis

Proteins were separated by SDS-PAGE and then electroblotted onto PVDF membranes (SequiBlot^TM^, Bio-Rad Laboratories, Inc., Hercules, CA, USA). After blocking with 3% (w/v) skim milk, the blots were incubated with rabbit antiserum (diluted to 0.01%) directed against purified HarA and cell-free extracts of strain C-4A at 4 °C for 15 h, and horseradish peroxidase-conjugated secondary antibodies at room temperature for 80 min.

The immunoreactive proteins were detected with an ECL western blot detection kit (GE Healthcare). An uncropped image of the blot is shown in Supplementary Figure 31.

### Derivatization of the reaction product

Harmaline was converted into the reaction product using cell-free extracts of *E. coli* BL21-CodonPlus(DE3)-RIL harboring pETDuet-*harA*. Derivatization of the reaction product was carried out as follows. The reaction mixture was incubated with 80 μg mL^−1^ DNPH (in acetonitrile) and 40 mM citrate buffer (pH 3.0) at 40 °C for 1 h. The derivatized reaction product was extracted with dichloromethane. The extract was evaporated and the residue was dissolved in 1 mL of acetonitrile.

### Structure determination of the reaction product

The derivative was purified by HPLC with a Shimadzu LC-10-Avp system equipped with a COSMOSIL πNAP 20 × 150 mm column (Nacalai Tesque) under the following conditions: column temperature, 40 °C; isocratic elution; mobile-phase composition, 50% deionized water/50% acetonitrile (v/v); flow rate, 6 mL min^−1^; and detection 500 nm. The purified derivative was evaporated and the residue was dissolved in DMSO-d_6_. The chemical structure of the derivative was elucidated by obtaining ^1^H NMR, ^13^C NMR, HMBC and HMQC spectra using an AVANCE-600 NMR spectrometer (Bruker, Billerica, MA, USA).

### Measurement of NH_3_

Measurement of NH_3_ via dansyl chloride derivatization: All procedures were performed in the dark or under low light. One mM harmaline was converted into the reaction product in 100 μL of the reaction mixture containing HarA at 28 °C. After the reaction, 20 μL of 2 M NaOH, 30 μL of a saturated NaHCO_3_ solution and 2 μL of 100 mM dansyl chloride were added to the reaction mixture, followed by incubation at 40 °C for 1 h. Ten microliters of 100 mM proline was then added to the reaction mixture, followed by further incubation at 40 °C for 30 min. After incubation, 100 μL of acetonitrile was added to the reaction mixture. The resulting derivative was analyzed immediately by LC/MS.

Measurement of NH_3_ via NBD-F derivatization: All procedures were performed in the dark or under low light. One millimolar harmaline was converted into the reaction product in 100 μL of the reaction mixture containing HarA at 28 °C. After the reaction, 80 μL of MeOH, 20 μL of 20 mM EDTA, and 70 μL of NBD-F (in acetonitrile) were added to the reaction mixture, followed by incubation at 60 °C for 1 h. The reaction mixture was then kept on ice. After addition of 270 μL of 50 mM HCl, the resulting derivative was analyzed immediately by LC/MS. NH_3_ was quantified by means of a standard curve obtained from the peak area of derivatized authentic NH_3_ in the negative ion mode.

### Measurement of H_2_O_2_

The reaction mixture comprising 0.45 mg mL^−1^ HarA, 20 mM Hepes-NaOH (pH 7.0), 1 mM harmaline, 1 mM *N*-ethyl-*N*-(2-hydroxy-3-sulfopropyl)-3,5-dimethoxyaniline, 1 mM 4-aminoantipyrine, and 50 U ml^−1^ horseradish peroxidase was incubated at 28 °C. H_2_O_2_ was quantified using the absorbance and extinction coefficient of the blue pigment derived from *N*-ethyl-*N*-(2-hydroxy-3-sulfopropyl)-3,5-dimethoxyaniline and 4-aminoantipyrine at 595 nm^43^.

### Measurement of O_2_

O_2_ concentrations were measured with an oxygen electrode (Hansatech Instruments Ltd., Norfolk, UK). The reaction was initiated by injecting the enzyme solution (1 mL) into an electrode cuvette and carried out at 28 °C.

### Site-directed mutagenesis

Site-directed mutagenesis of HarA was performed using a KOD-plus mutagenesis kit (Toyobo Co., Ltd., Osaka, Japan). The primers used in this experiment are listed in Supplementary Table 5. Plasmids containing each of the Y291A, D295A, N378A, Y379F, and H380A mutants of *harA* gene were transformed into *E. coli* BL21-CodonPlus(DE3)-RIL. The mutant enzymes were overexpressed and purified using the transformed *E. coli*. Purification procedures were the same as those described in Purification of the harmaline-metabolizing enzyme.

### Time courses of cell growth and enzymatic activity

Strain C-4A was cultured in 100 mL of liquid screening media containing harmaline as the sole carbon source for 72 h at 28 °C. The cells were inoculated 1% (v/v) into 1 L of liquid screening media containing each of harmaline or glucose as the sole carbon source, followed by incubation for 60 h at 28 °C. During cultivation, 10 mL aliquots of cultures were withdrawn at each check point for OD_600_ measurement, HPLC analysis of supernatants and preparation of cell-free extracts. The cell-free extracts were subjected to measurement of harmaline-degrading activity.

### Identification of topaquinone

Eight nmol of HarA was inactivated by the addition of 16 nmol of phenylhydrazine in two portions over a 1-h period. Using Amicon Ultra-10k (Merck Millipore, Burlington, MA, USA), excess phenylhydrazine was removed by exchanging the buffer with 100 mM Tris-HCl (pH 8.0). The derivatized enzyme was digested in a reaction mixture comprising 100 mM Tris-HCl (pH 8.0), 10 mM CaCl_2_, and 25 μg chymotrypsin (Roche Diagnostics, Basel, Schweiz). The reaction mixture was incubated at 25 °C for 4 h and then analyzed by LC/MS. The LC/MS conditions were as follows: flow rate, 1 mL min^−1^; solvent A, 0.05% (v/v) formic acid; solvent B, acetonitrile; and column, COSMOSIL πNAP 4.6 × 150 (Nacalai Tesque). After column equilibration with 100% solution A and 10 μl sample injection, a linear gradient system of acetonitrile, 0–100%, was applied over 60 min at 40 °C. LC/MS/MS fragmentation of an ion exhibiting a *m/z* value of 586 [M+2H]^+^ was conducted in the positive ion mode with collision energy of −20 mV.

### Resonance Raman spectroscopy

Resonance Raman analysis was carried out by 441.6 nm laser excitation (He-Cd laser (Kimmon Koha, IK4101R-F, Tokyo, Japan). The scattered light was dispersed with a single polychromator (Chromex, 500IS, Albuquerque, NM, USA), which was detected by a liquid-nitrogen-cooled CCD detector (Roper Scientific, Spec10:400B/LN, Trenton, NJ, USA). Indene was used to calibrate Raman shifts, giving an accuracy of ±1 cm^−1^ for intense Raman bands.

### Reporting Summary

Further information on experimental design is available in the Nature Research Reporting Summary linked to this article.

## Supplementary information

Supplementary Information

Source Data

Reporting Summary

## Data Availability

The authors declare that all the relevant data supporting the findings of the study are available in this article and its Supplementary Information files, or from the corresponding author upon request. The source data files underlying Figs. 1c–e, 2c–f, 3a, 4b–d, 5a and 6a, and Supplementary Figs. 2–5, 22, 24, 26 and 28b are provided as a Source Data file. The nucleotide sequence data reported in this paper appear in the DDBJ/GenBank database under accession numbers LC384995 for *harA*.
