# Supplementary material for: Copper amine oxidases catalyze the oxidative deamination and hydrolysis of cyclic imines
Source: Nat Commun. 2019 Jan 24;10:413. doi: 10.1038/s41467-018-08280-w (PMC6345859; doi:10.1038/s41467-018-08280-w)
Supplement: Supplementary file 1 — Supplementary Information [file 41467_2018_8280_MOESM1_ESM.pdf]

Supplementary Information

Copper amine oxidases catalyze the oxidative deamination and hydrolysis of cyclic imine

Nagakubo *et al.*

This file includes:

Supplementary Figures 1 to 31

Supplementary Tables 1 to 5

Supplementary Methods

Supplementary Notes

Supplementary Discussion

Supplementary References

Supplementary Figures

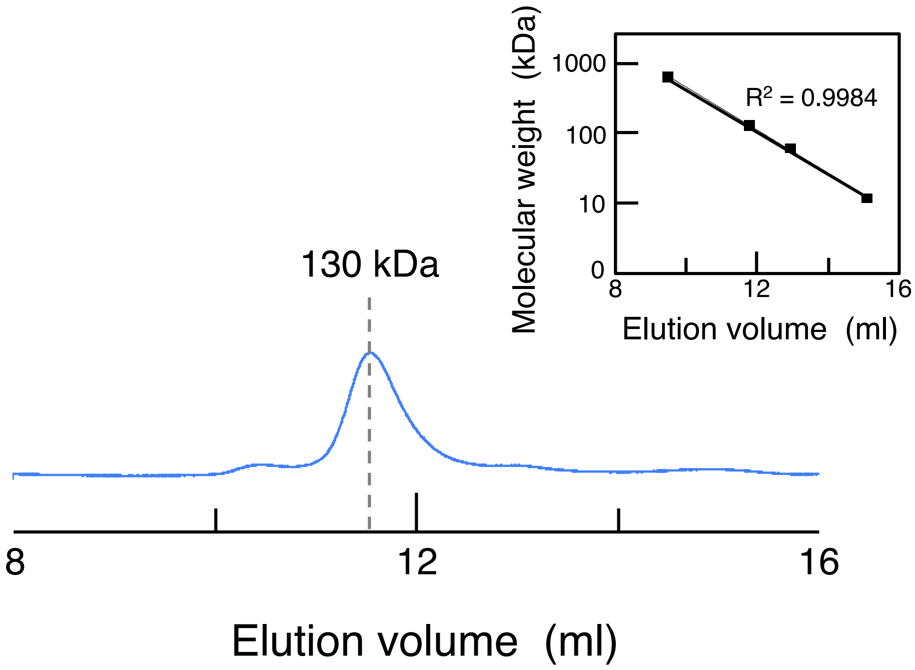

Supplementary Figure 1. Molecular mass determination of HarA.

Purified HarA was subjected to gel filtration to determine its molecular mass. The molecular mass determined is indicated by a dashed line. Inset: standard curve for calculation of molecular mass of HarA. Thyroglobulin (670 kDa), lactate dehydrogenase (142 kDa), enolase (67 kDa), and cytochrome *c* (12.4 kDa) were used as marker proteins.

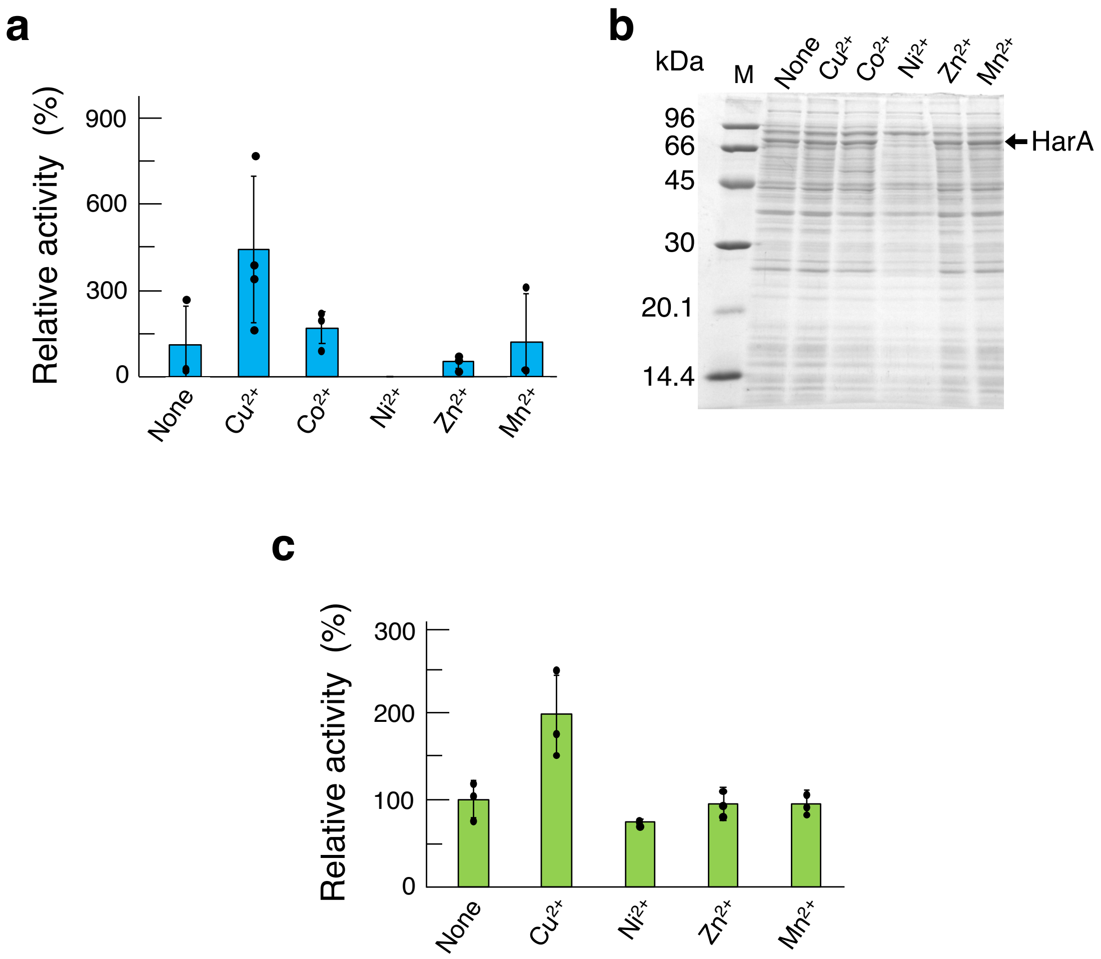

Supplementary Figure 2. HarA activities expressed in metal-supplemented media.

HarA activities of cell-free extracts of *E. coli* and strain C-4A were examined when they were cultivated in media containing each metal. (a) HarA activities of cell-free extracts of *E. coli* BL21-CodonPlus(DE3)-RIL cells harboring plasmid pETDuet-*harA* grown in metal-supplemented (5 μM each) M9 medium. HarA expression was induced by the addition of 0.1 mM isopropyl-β-D-thiogalactoside to the media. (b) SDS-PAGE of cell-free extracts used in *a*. (c) HarA activities of cell-free extracts of strain C-4A grown in metal-supplemented screening media containing harmaline as the sole carbon source. All data represent the mean values ± S.D. for at three experiments except for Cu^{2+} condition in *a*; this experiment was repeated four times. Source data are provided as a Source Data file.

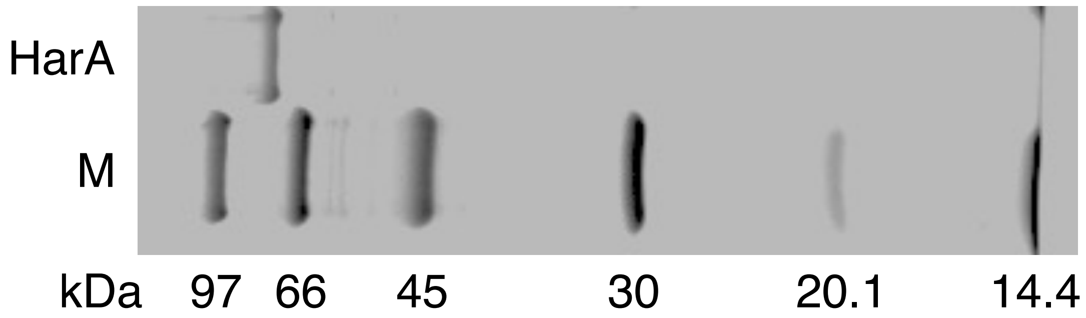

Supplementary Figure 3. The purified recombinant HarA.

Protein bands were detected by staining with Coomassie brilliant blue R-250. Lane M, marker proteins. Source data are provided as a Source Data file.

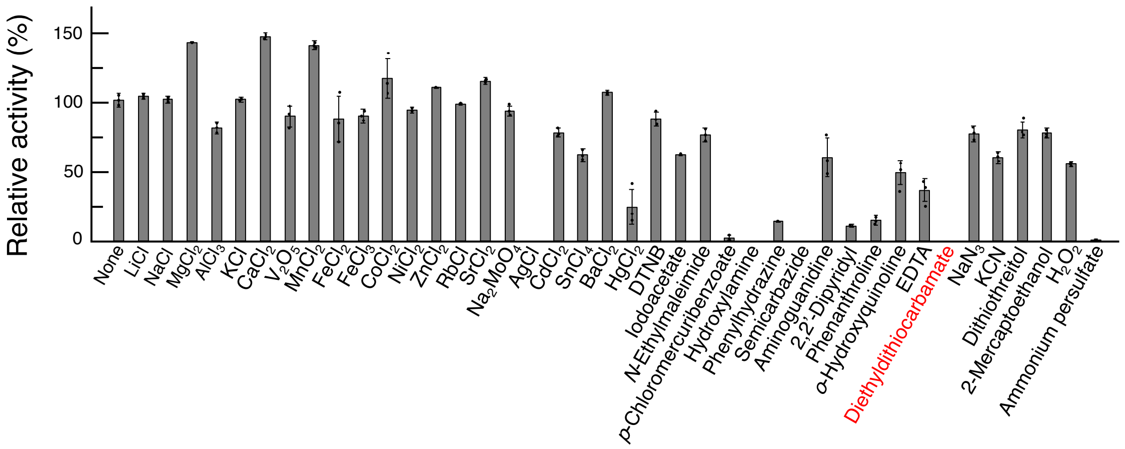

Supplementary Figure 4. Effects of various compounds on HarA activity.

Relative activities of HarA in the presence of various compounds are shown. Each compound was added to the reaction mixture containing HarA. Harmaline-degrading activity of HarA was then assayed after adding the substrate. The final concentrations of the tested compounds were as follows: metal salts, 20 or 100 μM; other tested compounds, 2 mM. All data represent the mean values ± S.D. for three experiments except for EDTA-added condition; this experiment was repeated four times. Source data are provided as a Source Data file.

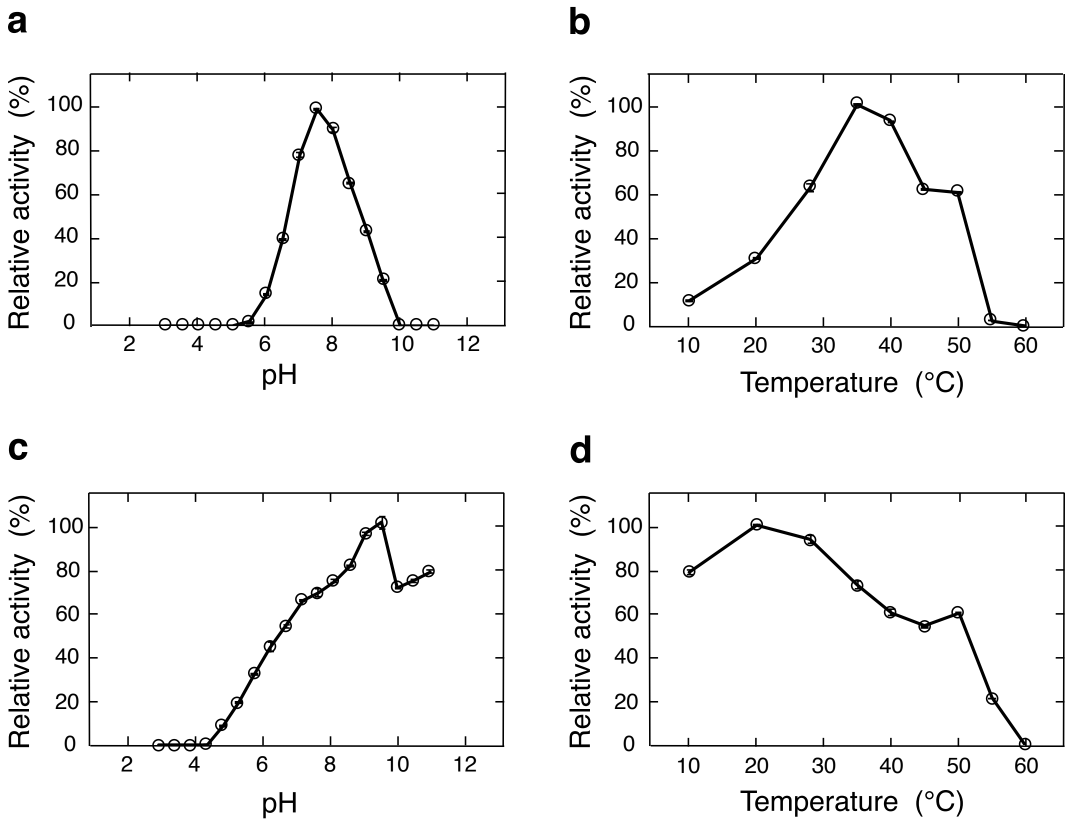

Supplementary Figure 5. Effects of temperature and pH on activity and stability of HarA.

Dependency and stability of HarA to pH and temperature were examined. (a) Reactions were carried out in Britton-Robinson buffer (pH 3-11). (b) Reactions were carried out at various temperatures. (c) HarA was incubated at various pH values at 20°C for 1 h in Britton-Robinson buffer at a concentration of 20 mM, an aliquot of each solution was taken, and then the activity of HarA was assayed under the standard assay conditions. (d) HarA was preincubated at various temperatures for 30 min in 20 mM Hepes-NaOH (pH 7.0), and then the residual activity was assayed. All data points represent the mean values ± S.D. for three experiments except for *a*; this experiment was repeated four times. Source data are provided as a Source Data file.

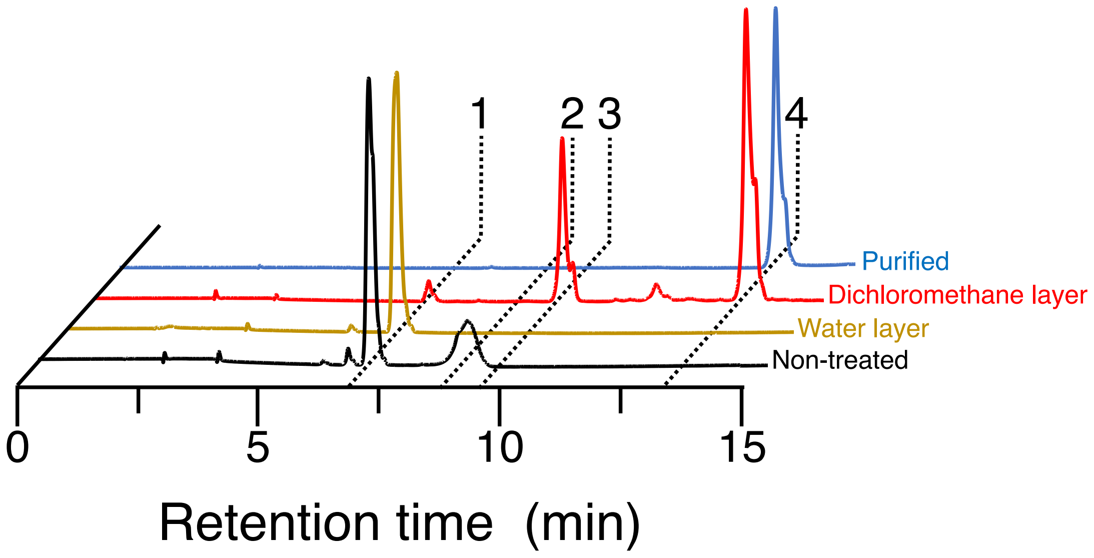

Supplementary Figure 6. Dinitrophenylhydrazine derivatization of reaction product.

2-AIMA was derivatized with dinitrophenylhydrazine (DNPH). Harmaline was converted into 2-AIMA by HarA (black). The resulting reaction mixture was subjected to dichloromethane extraction, and then separated into a water layer (yellow) and a dichloromethane layer (red). The dichloromethane layer was concentrated and purified by HPLC (blue). The numbers in the figure denotes the following compounds: 1, harmaline (MW: 214); 2, 2-AIMA (MW: 231); 3, DNPH (MW: 198); 4, DNPH-2-AIMA adduct (MW: 411).

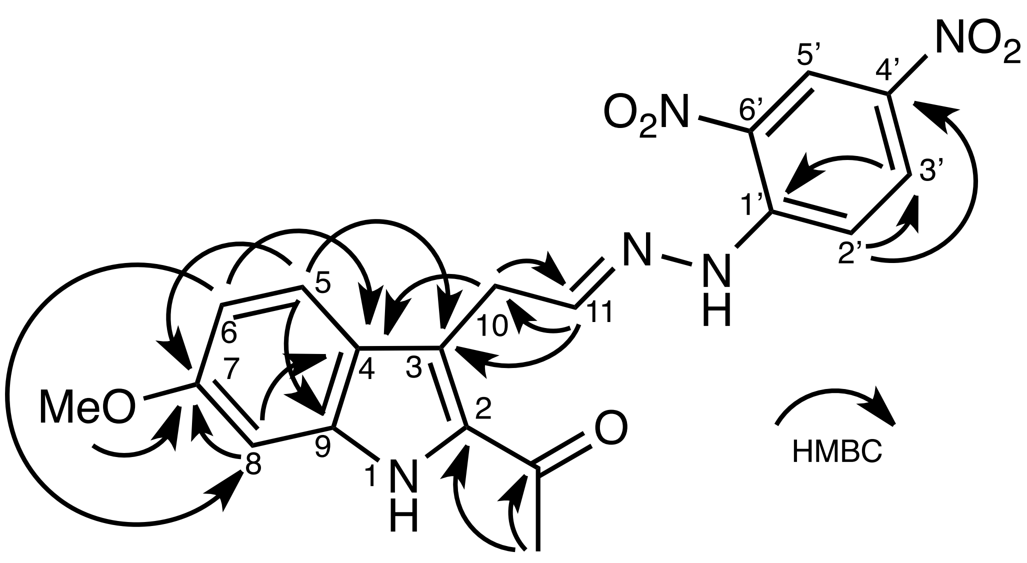

Supplementary Figure 7. HMBC correlations of 2-AIMA-DNPH adduct.

HMBC correlations for the determination of the structure of 2-AIMA-DNPH adduct. Arrows and numbers of atoms were assigned based on ^{1}H spectrum, ^{13}C spectrum, HMBC spectrum and HMQC spectrum of the compound.

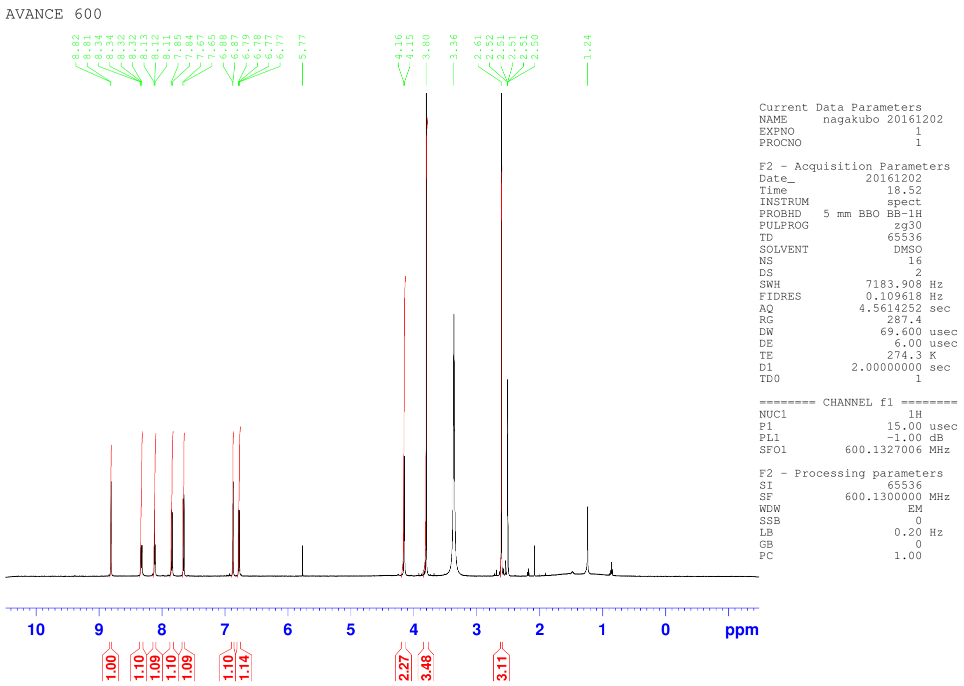

Supplementary Figure 8. **^{1}H spectrum of 2-AIMA-DNPH adduct.**

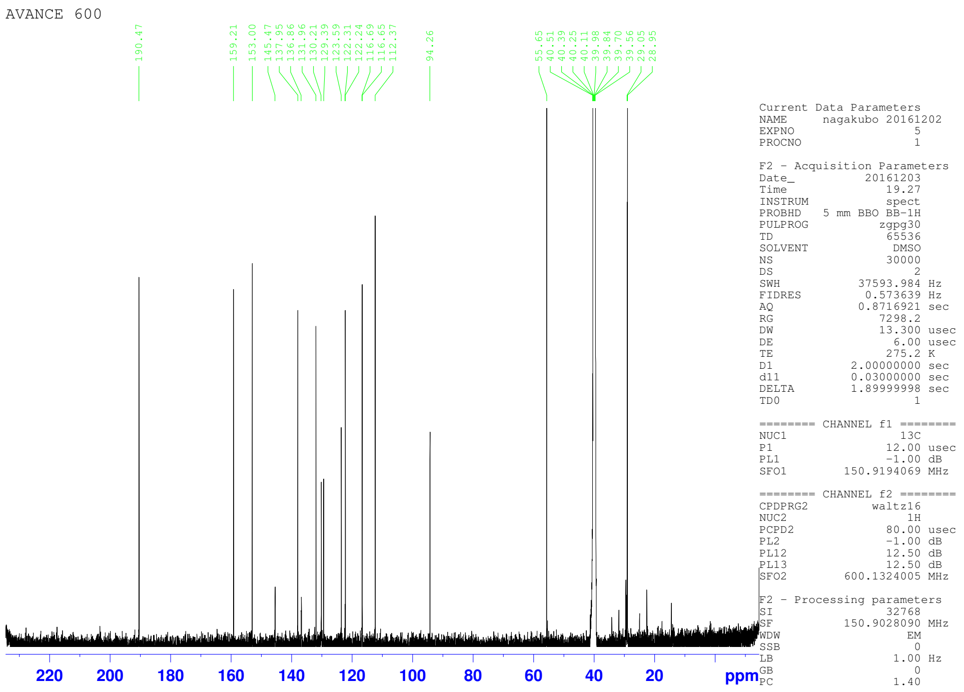

Supplementary Figure 9. ^{13}C spectrum of 2-AIMA-DNPH adduct.

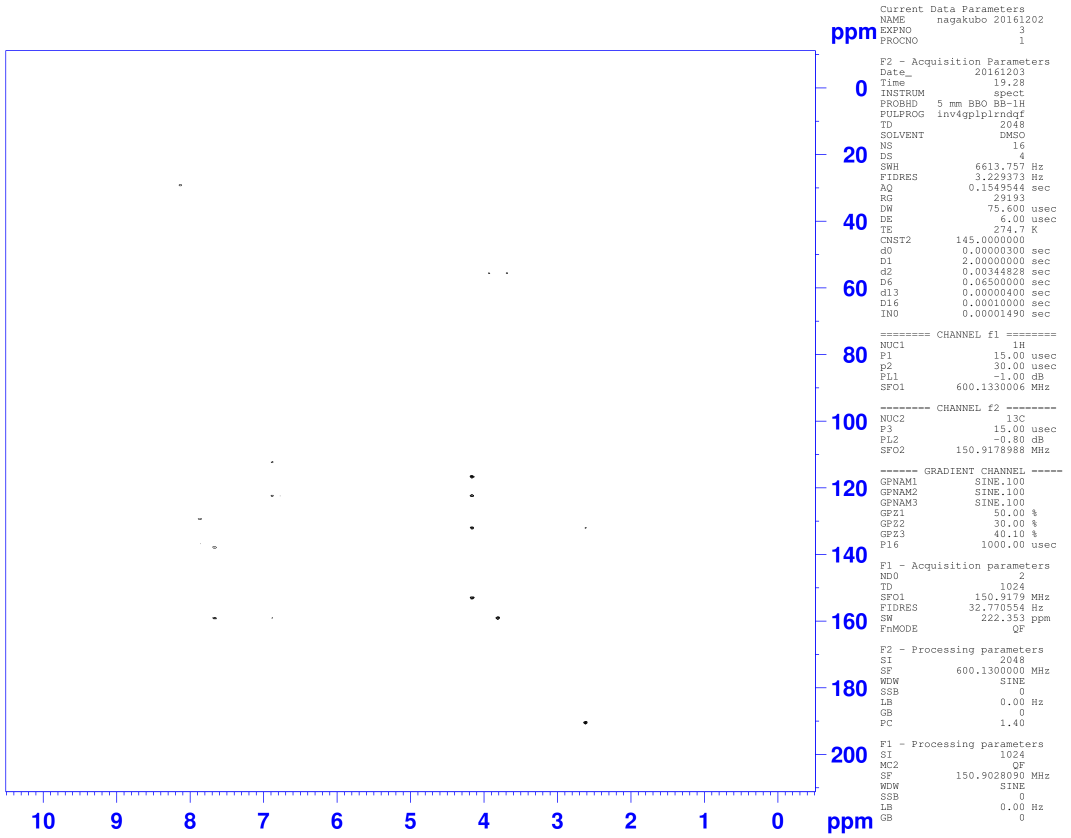

Supplementary Figure 10. **HMBC spectrum of 2-AIMA-DNPH adduct.**

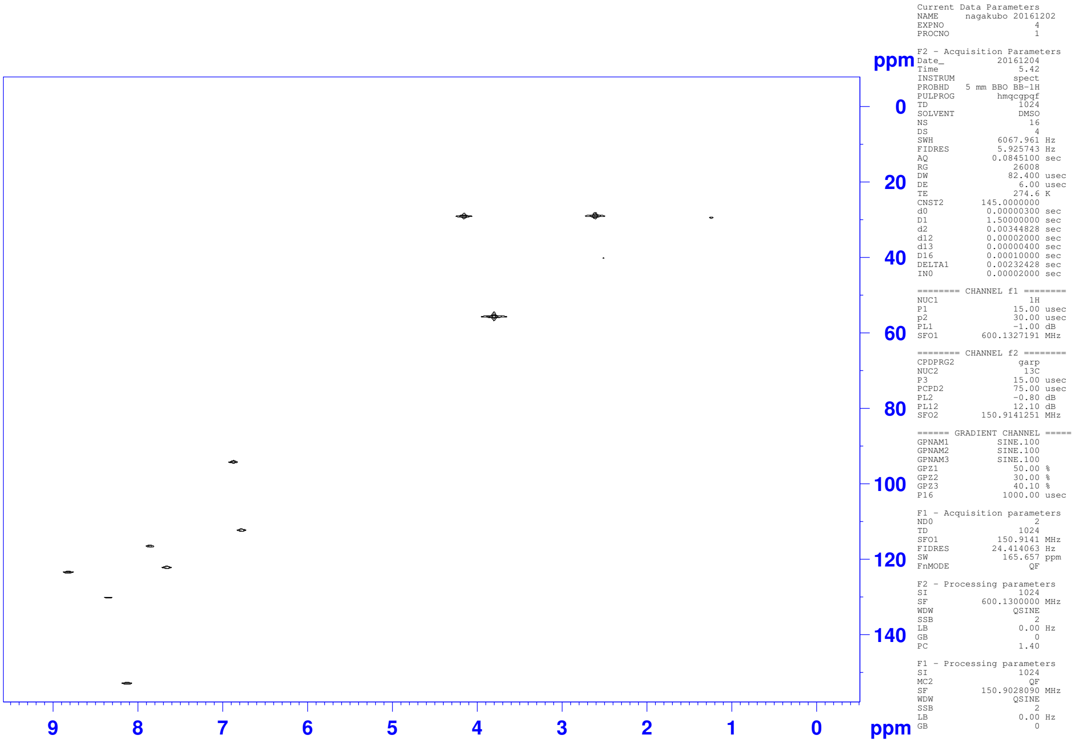

Supplementary Figure 11. HMQC spectrum of 2-AIMA-DNPH adduct.

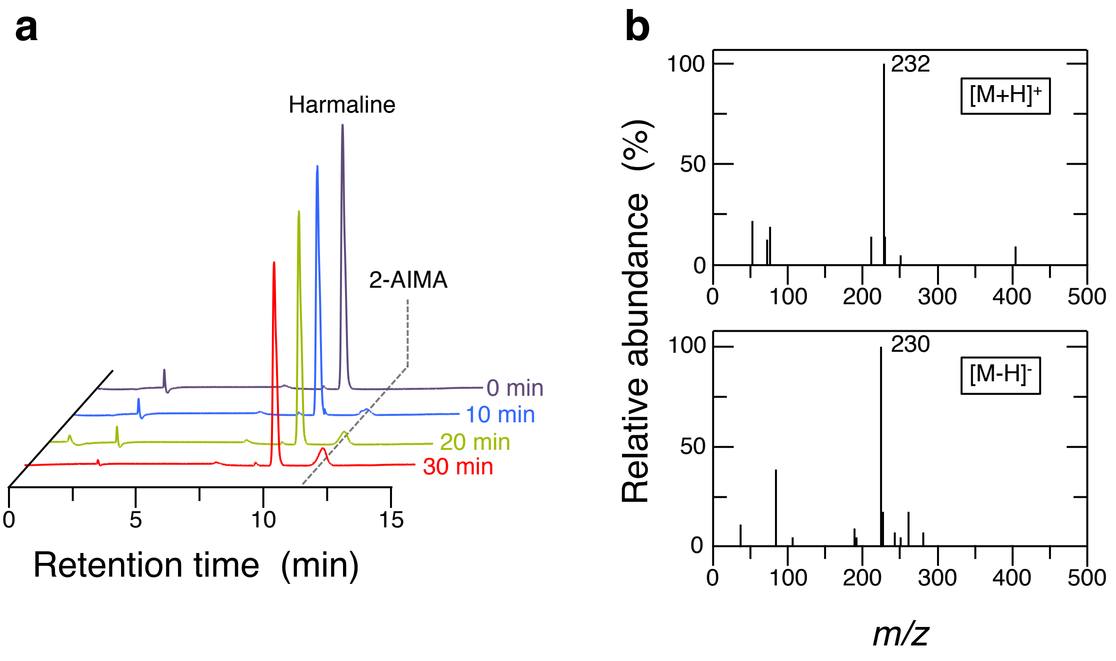

Supplementary Figure 12. Reaction specificity of HarA.

HarA formed no by-product during harmaline degradation. Harmaline consumed via the HarA-catalyzed reaction is completely converted into 2-AIMA. (a) The production of 2-AIMA through HarA-catalyzed harmaline degradation was analyzed by HPLC at 330 nm. (b) Mass spectra of the reaction product in *a* (30 min) are shown.

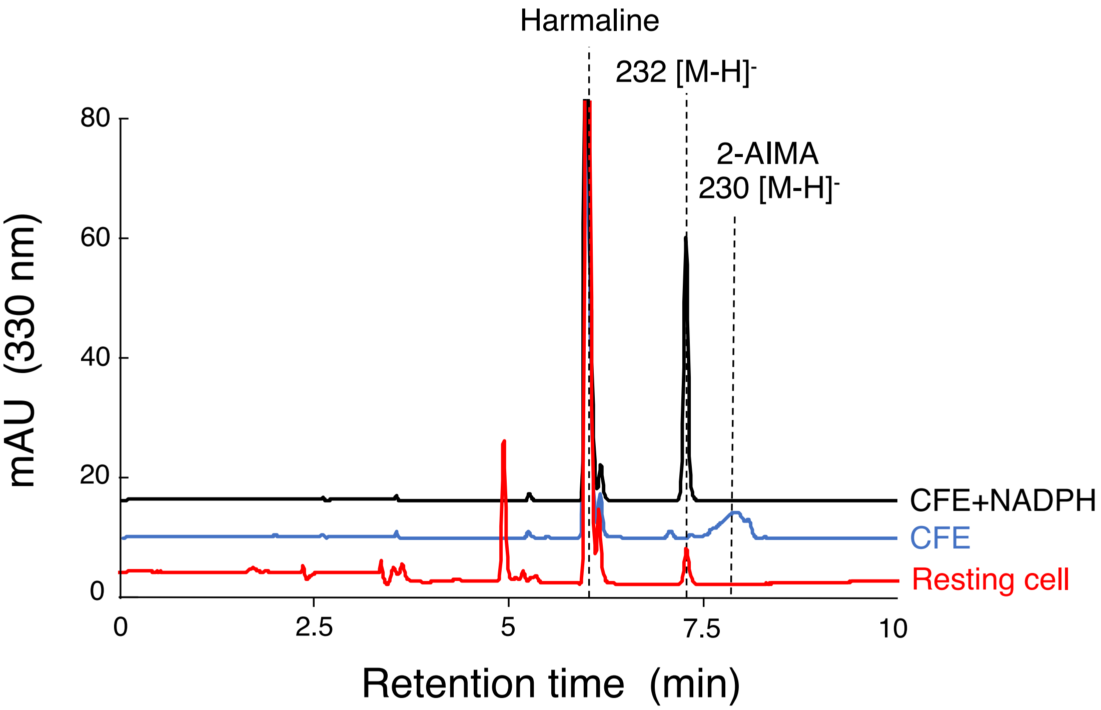

Supplementary Figure 13. Detection of a new metabolite which is derived from 2-AIMA.

2-AIMA was converted into a new metabolite by cell-free extracts of strain C-4A in the presence of NADPH. The reaction mixture containing cell free extracts of strain C-4A and harmaline was incubated for 60 min (blue). NADPH was then added to the above reaction mixture and incubated for 30 min again (black). The reaction mixture containing resting cells of strain C-4A was incubated with harmaline for 60 min (red).

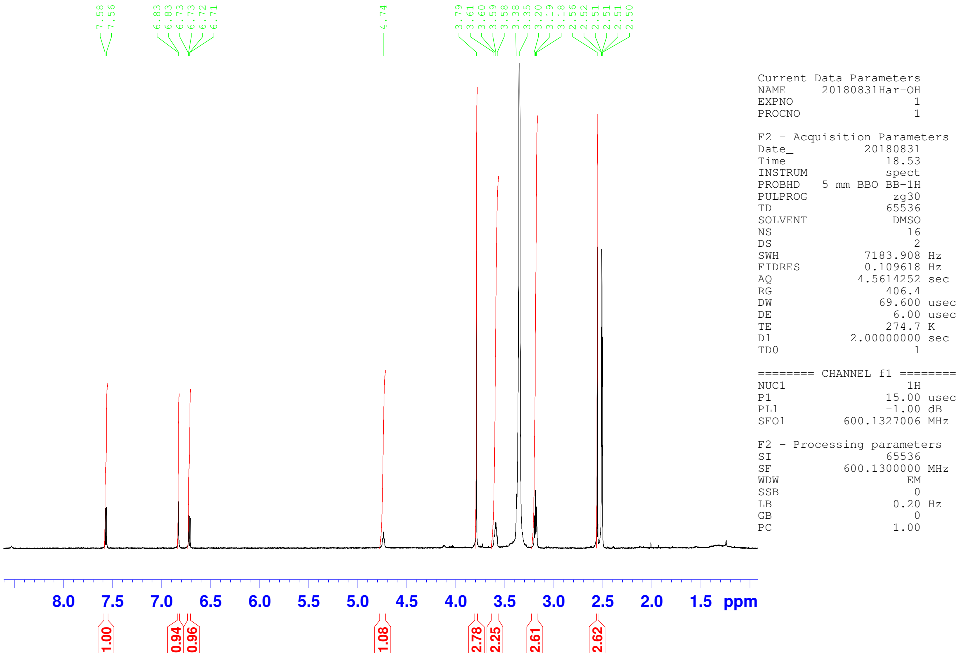

Supplementary Figure 14. **^{1}H spectrum of an alcohol derivative of 2-AIMA.**

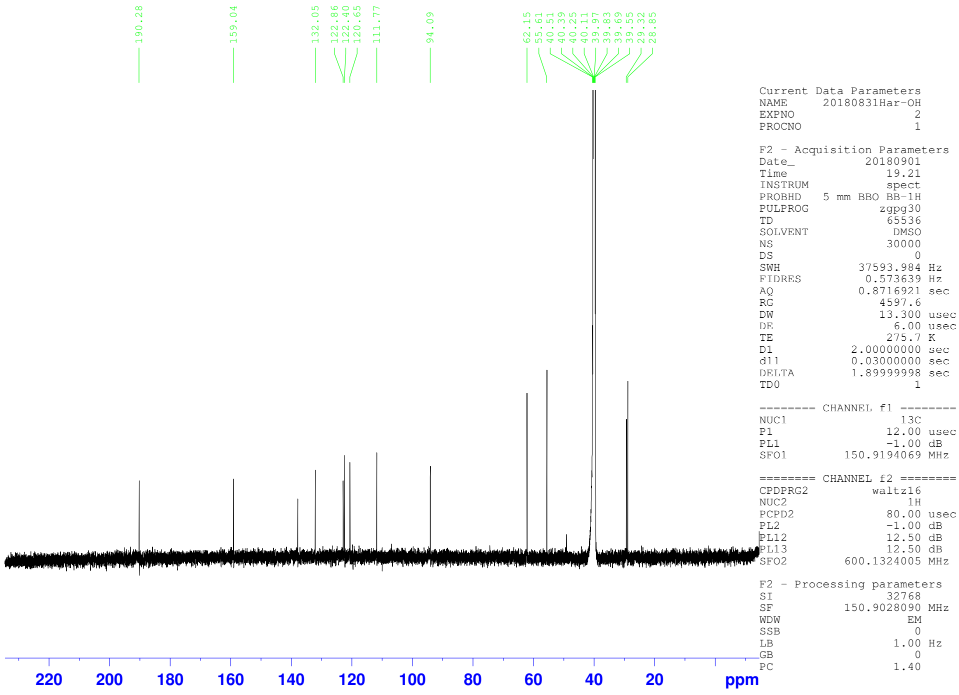

Supplementary Figure 15. **^{13}C spectrum of an alcohol derivative of 2-AIMA.**

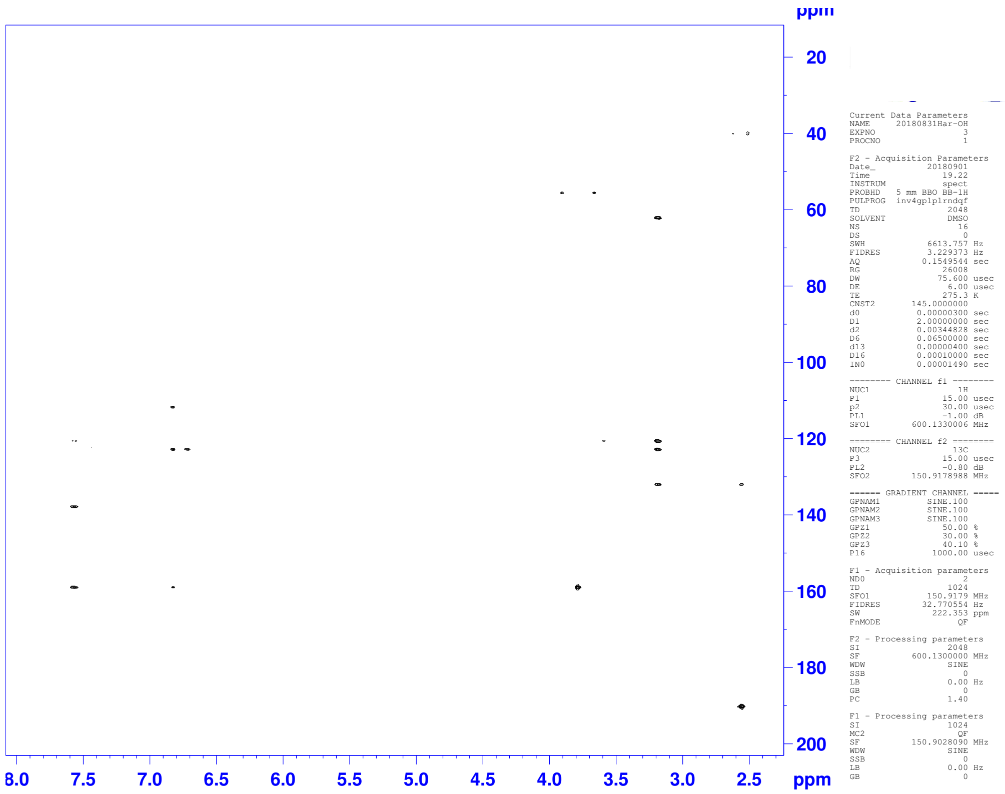

Supplementary Figure 16. HMBC spectrum of an alcohol derivative of 2-AIMA.

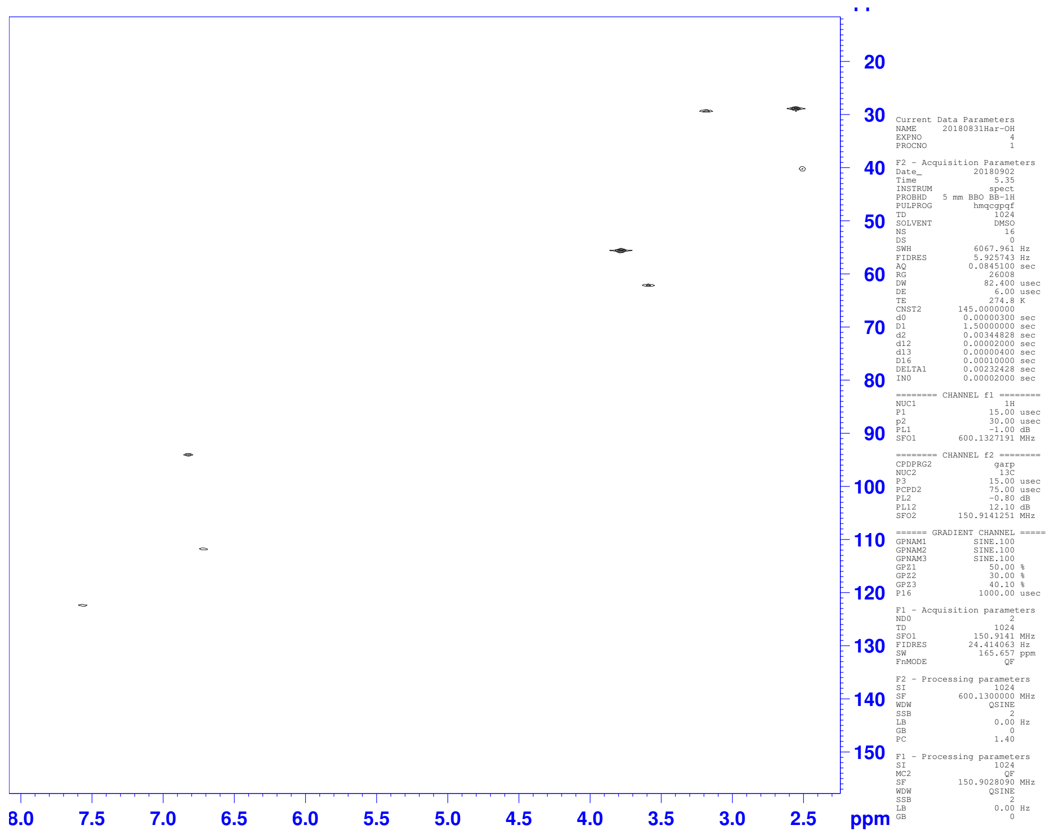

Supplementary Figure 17. HMQC spectrum of an alcohol derivative of 2-AIMA.

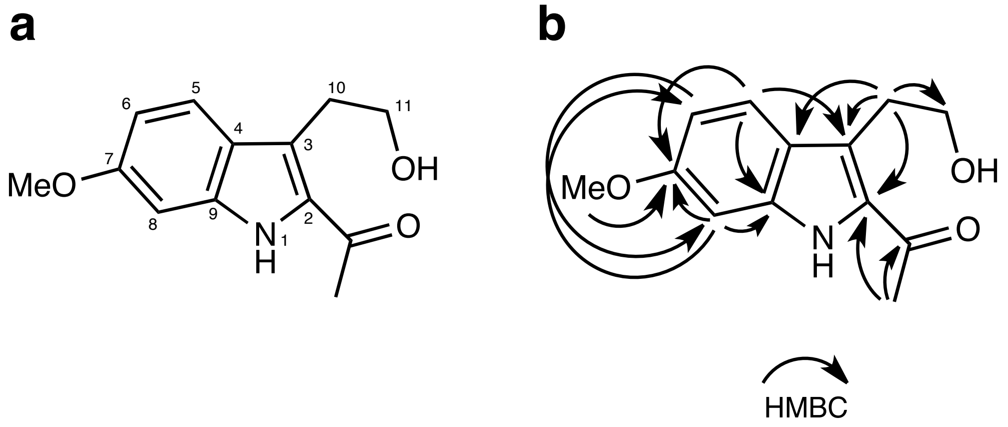

Supplementary Figure 18. Determined structure and HMBC correlations of an alcohol derivative of 2-AIMA.

HMBC correlations for the determination of the structure of an alcohol derivative of 2-AIMA. (a) Numbers of atoms and (b) arrows were assigned based on ^{1}H spectrum, ^{13}C spectrum, HMBC spectrum and HMQC spectrum of the compound.

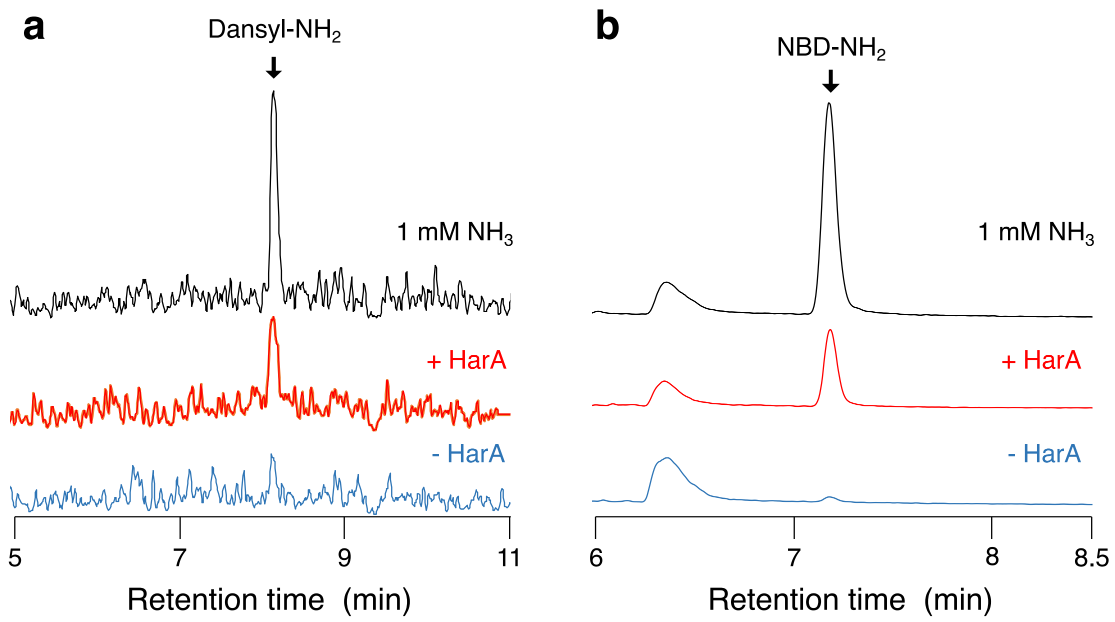

Supplementary Figure 19. NH_3 formation during harmaline degradation by HarA.

During HarA-catalyzed harmaline degradation, formation of NH_3 was detected by LC/MS. (a) Mass chromatograms of *m/z* 251, which corresponds to NH_3 adduct of dansyl chloride (dansyl-NH_2), in the positive ion mode. (b) Mass chromatograms of *m/z* 179, which corresponds to NH_3 adduct of NBD-F (NBD-NH_2), in the negative ion mode.

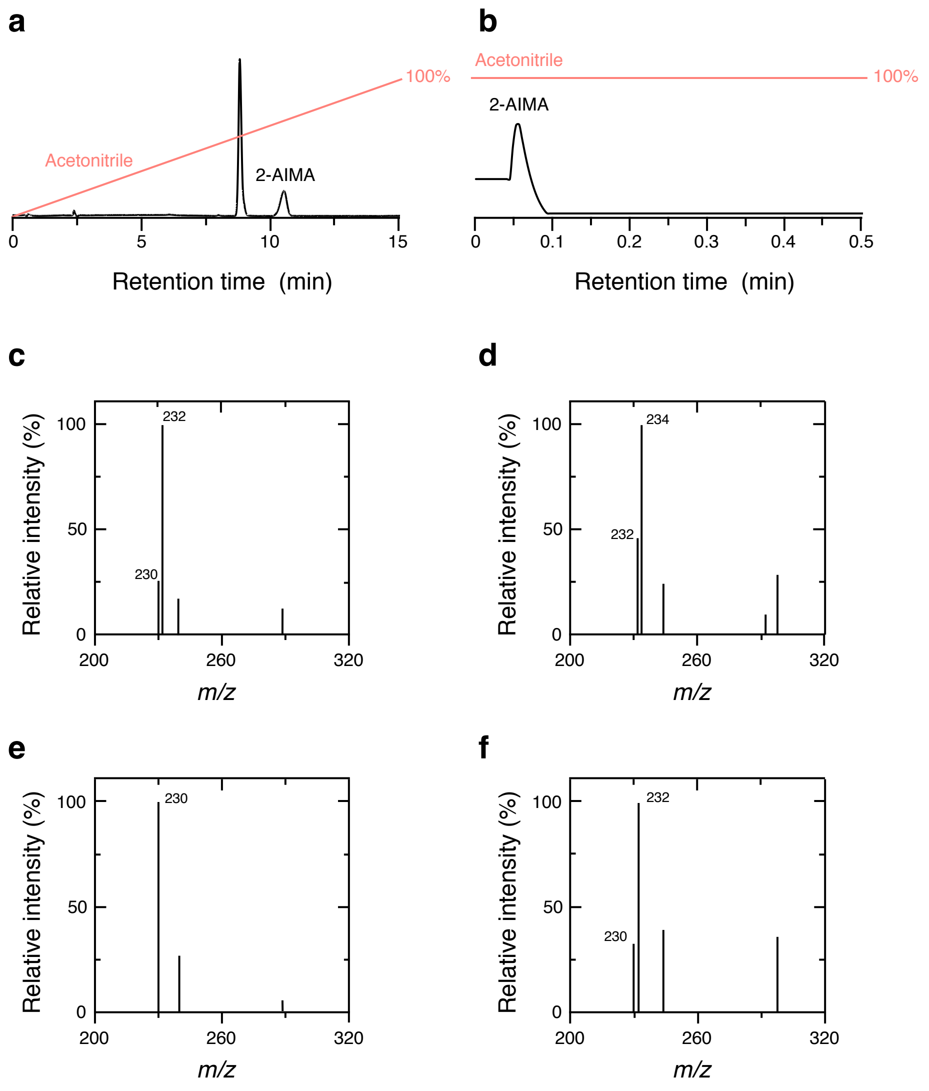

Supplementary Figure 20. Detection of double ^{18}O-labeled 2-AIMA on LC/MS.

2-AIMA was ^{18}O-labelled using $H_2{}^{18}O$ and then analyzed by LC/MS. (a) The standard reaction mixture containing 90% $H_2{}^{18}O$ was analyzed by LC/MS in linear gradient mode. (b) An ethyl acetate extract of the above mixture was analyzed by LC/MS in isocratic mode. The peaks corresponding to 2-AIMA are indicated in *a* and *b*. (c) MS spectrum of 2-AIMA detected in *a*. (d) MS spectrum of 2-AIMA detected in *b*. *m/z* values of 230, 232 and 234 correspond to non-labeled, single ^{18}O-labeled and double ^{18}O-labeled 2-AIMA, respectively. (e,f) 2-AIMA which was enzymatically synthesized by HarA in $H_2{}^{16}O$ was diluted in $H_2{}^{18}O$ ($H_2{}^{16}O$: $H_2{}^{18}O$ = 3 : 7) after removal of the enzyme by ultrafiltration, extracted by ethylacetate, and analyzed by LC/MS in the isocratic mode shown in *b*. MS spectra of 2-AIMA before and after dilution in $H_2{}^{18}O$ are shown in *e* and *f*, respectively.

a

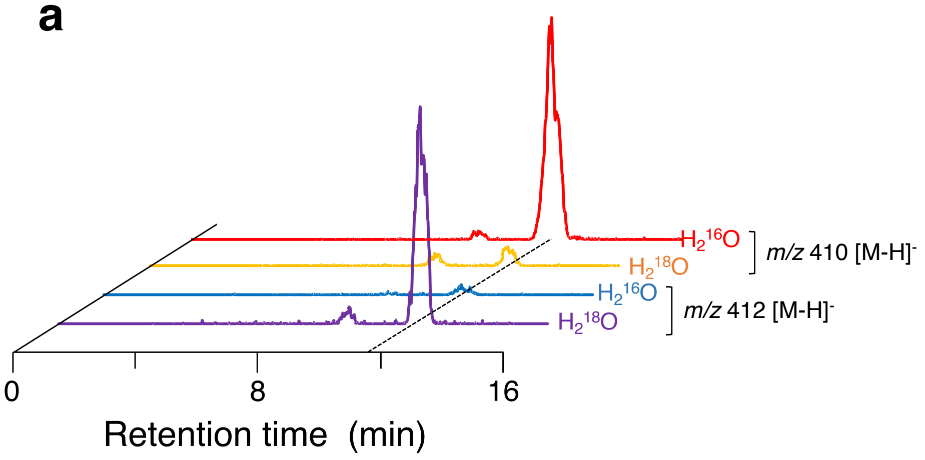

b

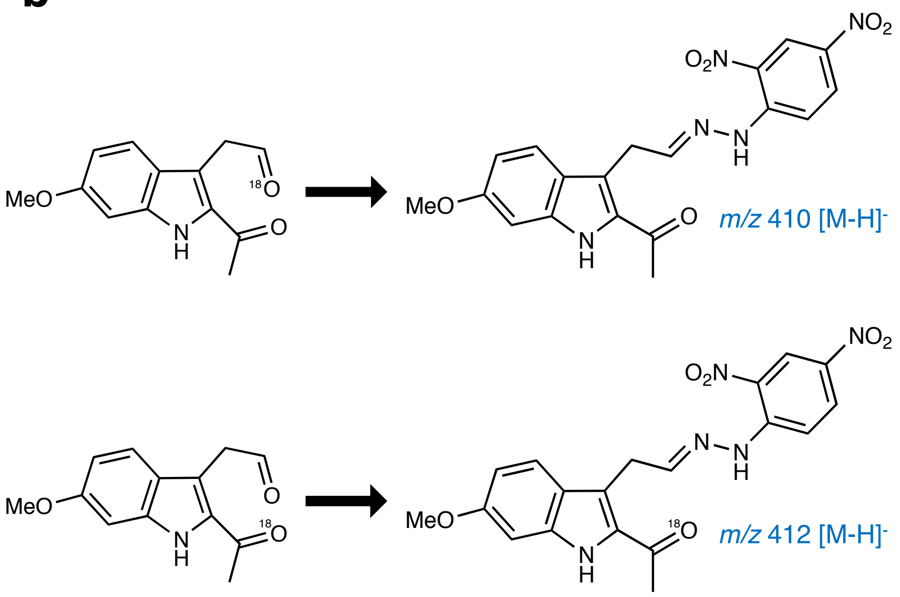

Supplementary Figure 21. Incorporation of an oxygen atom derived from H_2O into 2-AIMA.

^{18}O-labelling and DNPH derivatization of 2-AIMA were performed. (a) After producing 2-AIMA through HarA-catalyzed harmaline degradation in $H_2{}^{16}O$ or $H_2{}^{18}O$, each of the reaction products was derivatized with DNPH, and then analyzed by LC/MS. Mass chromatograms of *m/z* 410 and 412 in the negative ion mode are shown. (b) To determine which of the two carbonyl groups was labelled by $H_2{}^{18}O$, ^{18}O-labelled 2-AIMA was derivatized with DNPH.

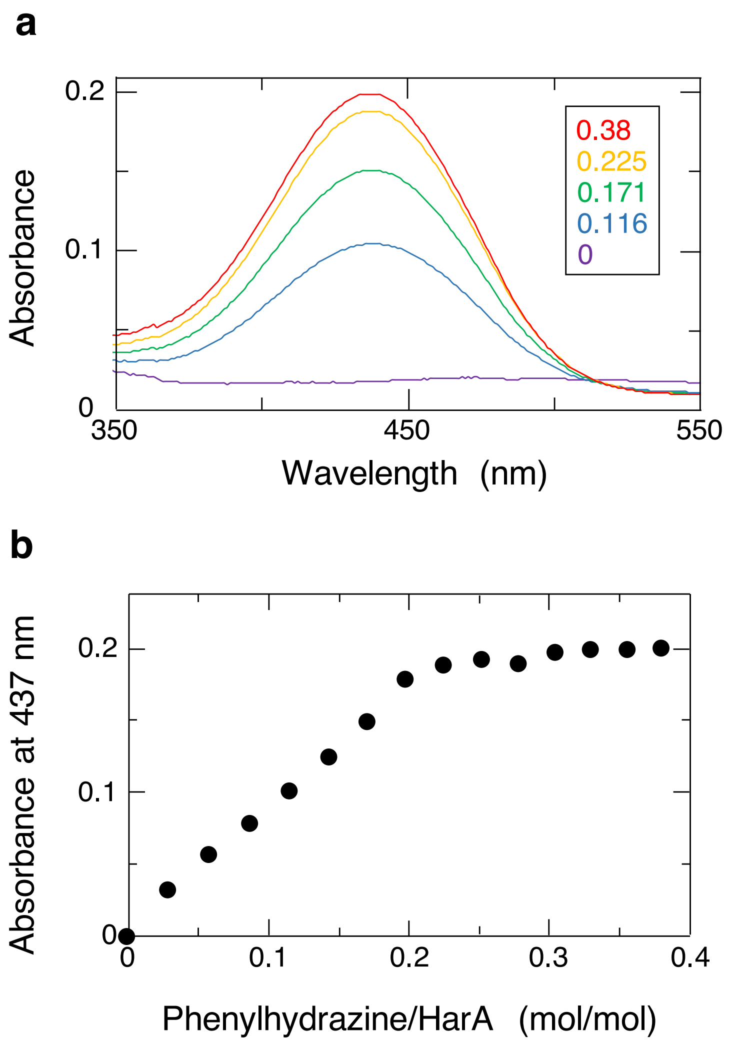

Supplementary Figure 22. Topaquinone quantitation using pheynylhydrazine.

TPQ-phenylhydrazine adduct was spectrophotometrically detected and quantified. (a) Spectroscopic detection of the TPQ-phenylhydrazine adduct. Final concentration of HarA was 1 mM. Inset: Final concentrations (mM) of added phenylhydrazine. (b) Phenylhydrazine titration of TPQ to determine the phenylhydrazine/HarA subunit ratio. Source data are provided as a Source Data file.

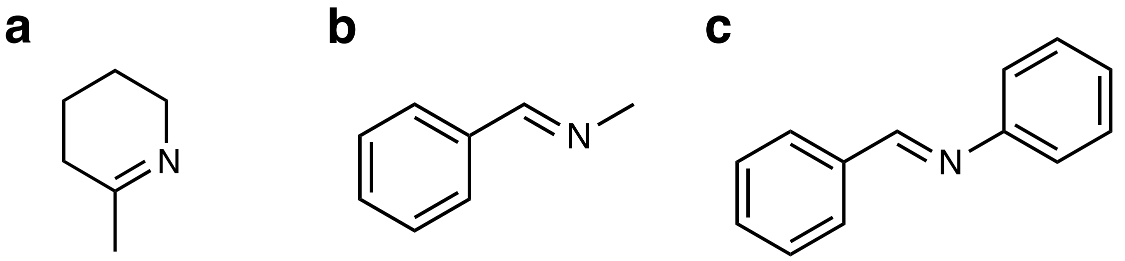

Supplementary Figure 23. Compounds used for examination of substrate specificity of HarA.

These compounds were tested as substrates for HarA. (a) 2-Methylenepiperidine. (b) *N*-Benzylidenemethylamine. (c) *N*-Benzylideneaniline.

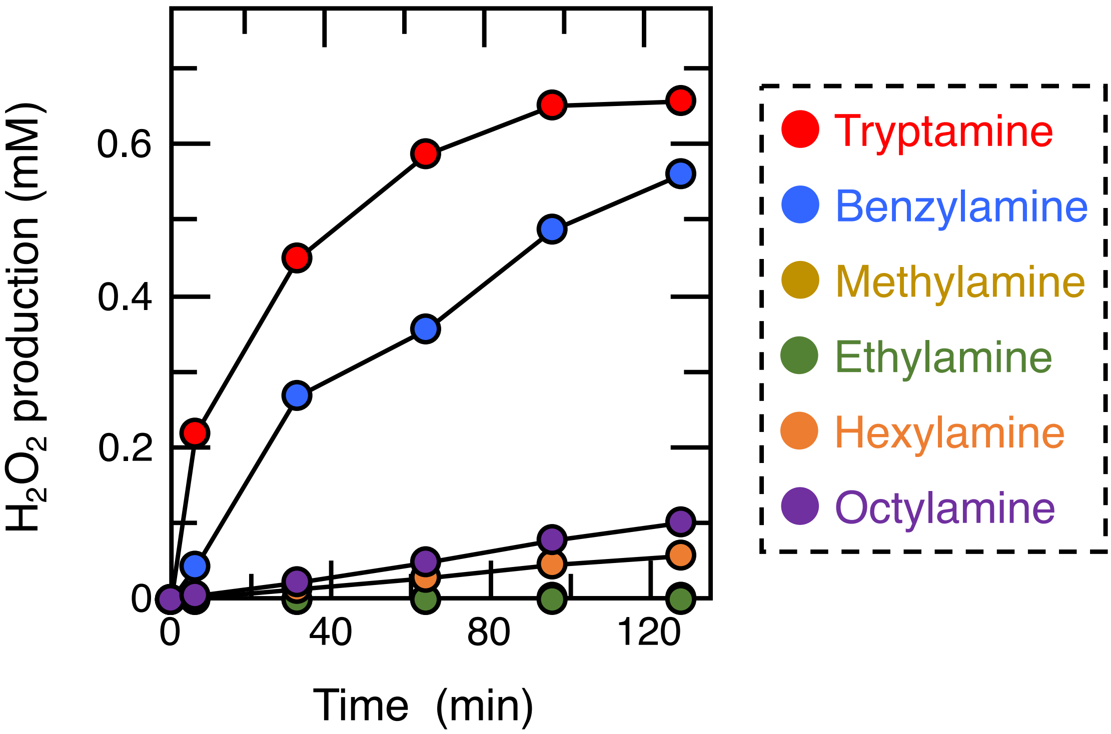

Supplementary Figure 24. Substrate specificity of HarA toward amine substrates. Each amine substrate was incubated with 0.09 mg ml^{-1} HarA. The concentration of each substrate was 1 mM. H_2O_2 production during reactions was measured by the DAOS method (described under Methods). Source data are provided as a Source Data file.

a

```
HarA  6     FDSLTADEITMVSTLIKQKGIGGERPGFGSVFTHEPDK----ARLRRGDKVTRQARALVL
             ++LTADEI     ++K          F  +    PDK    A       V +  +A V+
ECAO  96    LNALTADEIKQAVEIVKASADFKPNTRFTEISLLPPDKEAVWAFALENKPVDQPRKADVI

      62    DRNSAATFDVIVDLEAEAIASVVQLTDGGAQMLAEEIELADQIAKASAEYIEALSKRGIT
              +     + +VDL+   + S   + D    +L ++      I   S E+  A+ KRGIT
      156   MLDGKHIIEAVVDLQNNKLLSWQPIKDAHGMVLLDDFASVQNIINNSEEFAAAVKKRGIT

      122   DLSLVQLDPFGVGNRGDID--VEGRRLWACVSYFRHFEDDNAYAHIIEGVIAIVDTVRCE
            D   V   P  VG     D   +  RL   +SY     D N +AH IE ++A+VD  + +
      216   DAKKVITTPLTVGYFDGKDGLKQDARLLKVISYL-DVGDGNYWAHPIENLVAVVDLEQKK

      180   VYAIEDTGVKPMPMTCNNYTADANGPMREDVKRLDILQVDGPGFTLDGPQMKWQKWRFRI
            +  IE+  V P+PMT   +  D    +   VK + I++ +G  +T+ G  + W+ W F
      275   IVKIEEGPVVPVPMTARPF--DGRDRVAPAVKPMQIIEPEGKNYTITGDMIHWRNWDFHL

      240   NMHPIDGLVLSGIEYDDSKAGQPENYRSVMHRASLAEMIVPYGIPEAAHYFRSAFDAGEY
            +M+   G ++S + Y+D+        R VM+  SL  MIVPYG P+   YF++  D+G+Y
      333   SMNSRVGPMISTVTYNDNGTK-----RKVMYEGSLGGMIVPYGDPDIGWYFKAYLDSGDY

      300   GLGKMANSLVLGCDCLGDITYLDAVMADDDGNPALIKNAICIHEEDAGILWKHTDWATGK
            G+G + + +  G D   +   L+  +AD  G P  I  AI + E  AG  +KH +
      388   GMGTLTSPIARGKDAPSNAVLLNETIADYTGVPMEIPRAIAVFERYAGPEYKHQEMGQPN

      360   VDVRRSRKLVVSFIATIGNYHYGFYWNFFQDGHIEVDTKLMGV-----VQTISYEDGQVP
            V   R R+LVV +I+T+GNY Y F W F ++G I +D    G+     V+  +  D
      448   VSTER-RELVVRWISTVGNYDYIFDWIFHENGTIGIDAGATGIEAVKGVKAKTMHDETAK

      415   PNA---SPIGENLAATWHQHLFNFRLDMEVDGWQNSVYQNDVDGEPVGPANPYGNAIRAT
             +    + I  N+  T HQH++NFRLD++VDG  NS+   D   +P     P  + ++
      507   DDTRYGTLIDHNIVGTTHQHIYNFRLDLDVDGENNSLVAMDPVVKPNTAGGPRTSTMQVN

      472   KTLIAREQDGDGLANPQTARSWTVANPNKHNAWGVPVGYKLLPGWASDTLIAQEPSL---
            +  I  EQD     +P T R   ++NPNK N  G PV Y+++P       +A+
      567   QYNIGNEQDAAQKFDPGTIR--LLSNPNKENRMGNPVSYQIIPYAGGTHPVAKGAQFAPD

      529   --MVKRAGFATKNMWVTPYAPDEMHSAGDHPNQDRAGAGLPAWTAANRPVENTDVVLWHT
              +  R  F  K +WVT Y P E    G +PN+     GL  ++  N  ++NTD V+W T
      625   EWIYHRLSFMDKQLWVTRYHPGERFPEGKYPNRSTHDTGLGQYSKDNESLDNTDAVVWMT

      587   VGVTHIPRSEDWPVMPNETASFMLVPNNFFDKNPAL  622
             G TH+ R+E+WP+MP E    +L P NFFD+ P L
      685   TGTTHVARAEEWPIMPTEWVHTLLKPWNFFDETPTL  720
```

b

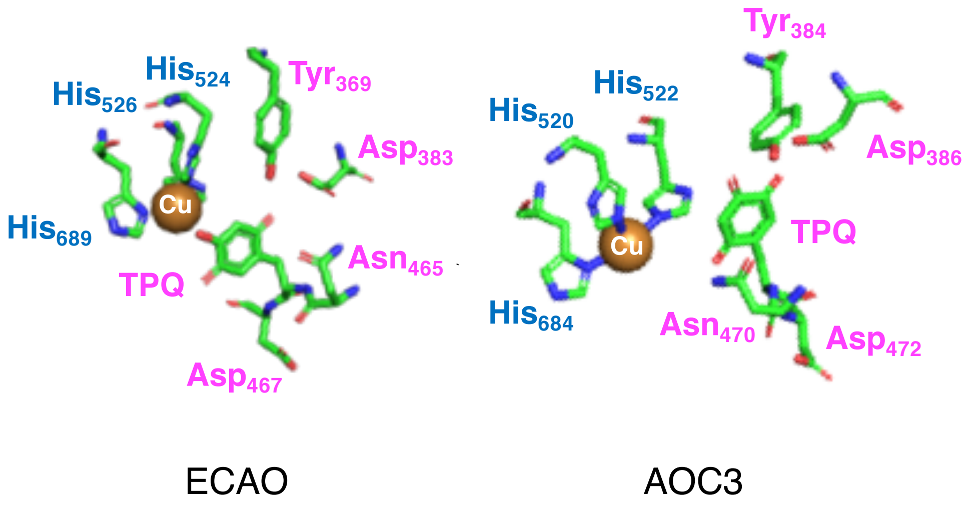

Supplementary Figure 25. Amino acid sequence alignment of HarA and *E. coli* copper amine oxidase.

Comparison of amino acid sequences and X-ray crystallographic structures among CAOs. (a) Sequence alignment of HarA with CAO from *E. coli* (ECAO). Amino acid residues surrounding TPQ (magenta) or copper (blue) are indicated. (b) Active site arrangements of ECAO (Protein Data Bank [PDB] entry 1OAC [https://doi.org/10.2210/pdb1OAC/pdb]; left) and human AOC3 (PDB entry 3ALA[https://doi.org/ 10.2210/pdb3ALA/pdb]; right).

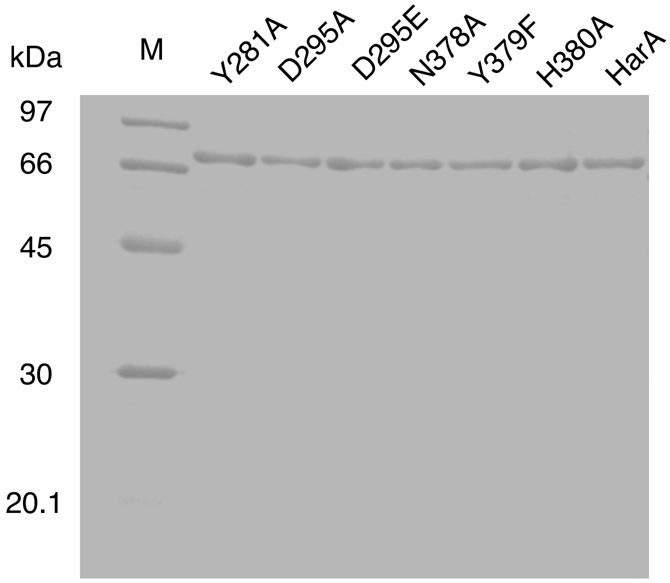

Supplementary Figure 26. The purified HarA mutants.

SDS-PAGE of HarA mutants used in this study. Lane M, marker proteins. Source data are provided as a Source Data file.

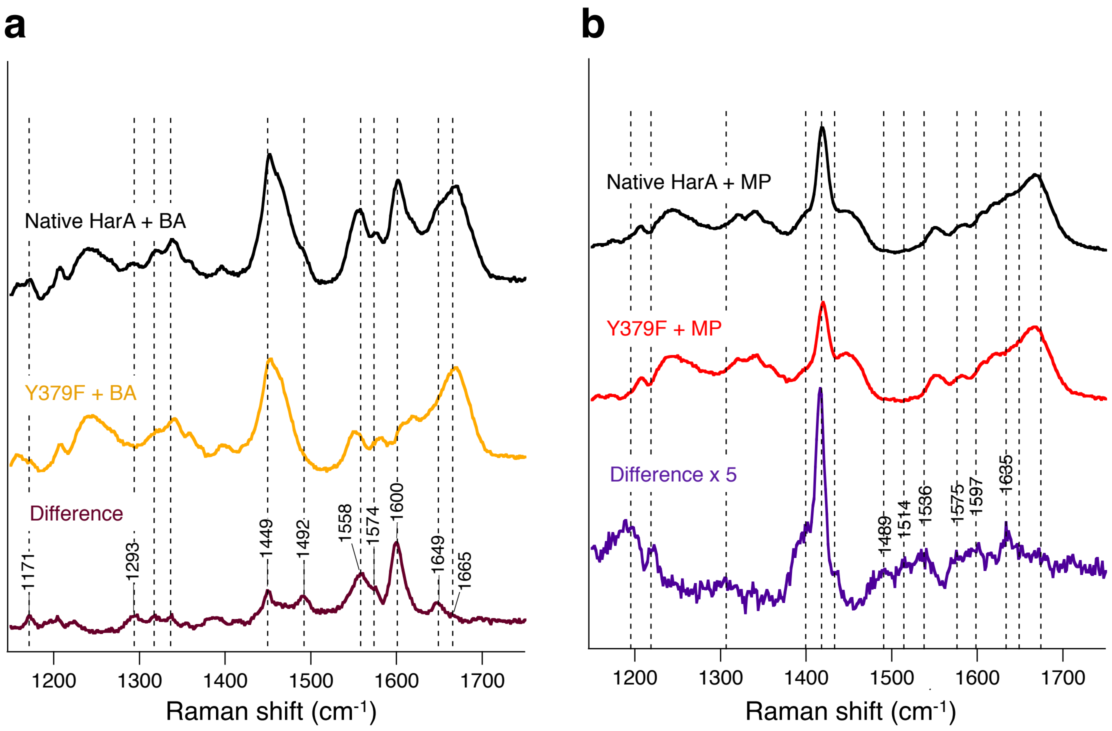

Supplementary Figure 27. Raman spectra of reaction intermediates of HarA.

Raman measurements were performed on HarA with an amine (benzylamine) or imine (methylenepiperidine) substrate in an anaerobic environment. HarA was added to the reaction mixture containing (a) 13 equivalents of benzylamine (BA) or (b) 5 equivalents of 2-methylenepiperidine (MP).

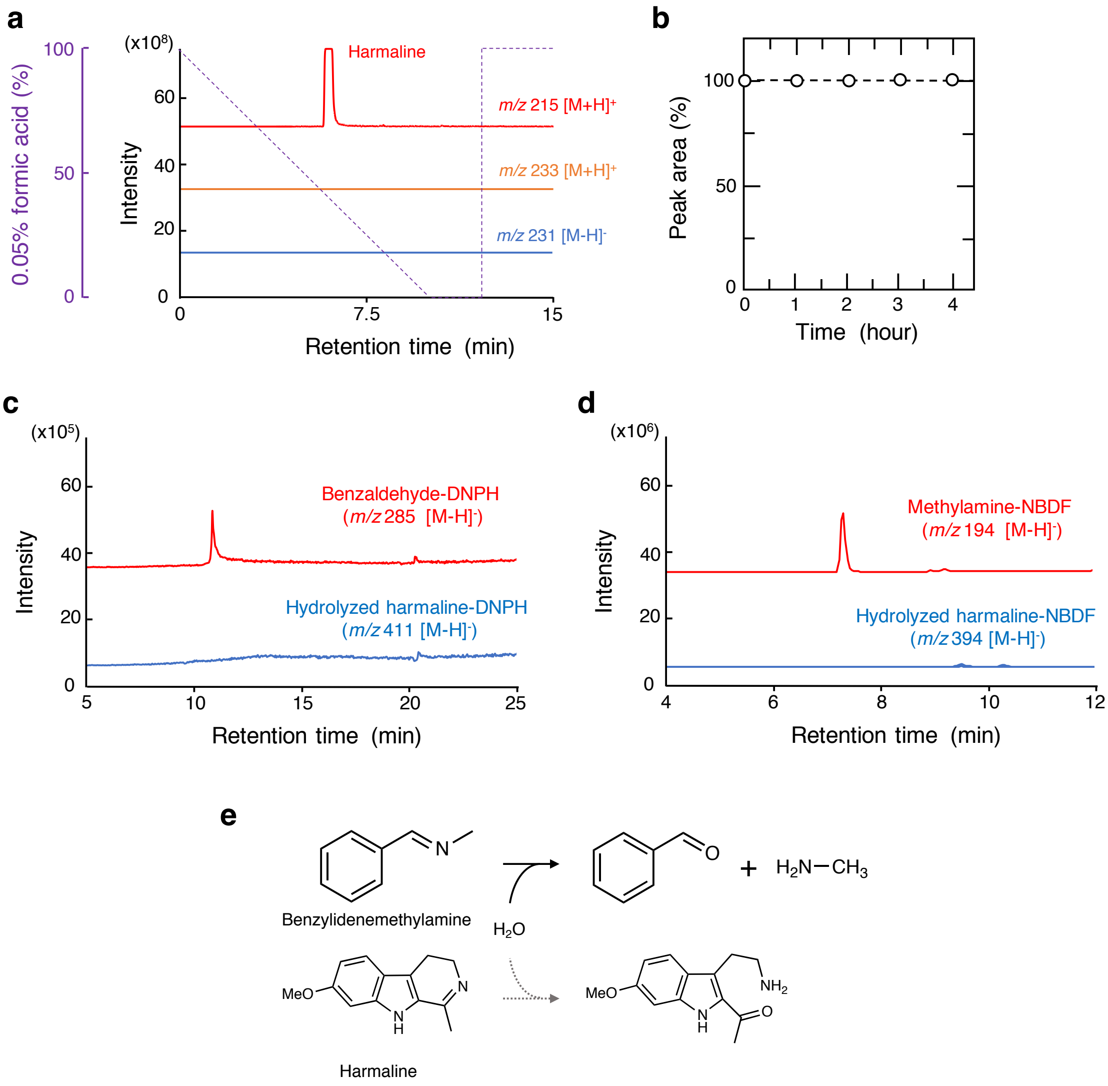

Supplementary Figure 28. Stability of cyclic imine within harmaline.

Stability of harmaline in water was examined. (a) Hydrolytic products of harmaline (orange and blue) in 20 mM Hepes-NaOH (pH 7.0) were analyzed by LC/MS in selective ion mode after 2 h incubation at 28°C. Carriers were 0.05% formic acid and acetonitrile. (b) Peak area of harmaline at *m/z* 215 $[M+H]^+$ in the negative ion mode of LC/MS was measured during incubation of the above solution. Hydrolytic products of benzylidenemethylamine (linear imine) and harmaline (cyclic imine) were derivatized by (c) DNPH and (d) NBD-F, and analyzed by LC/MS as described under Methods. Concentrations of each compounds were 1 mM. The structures of possible hydrolytic products of the imines are shown in *e*.

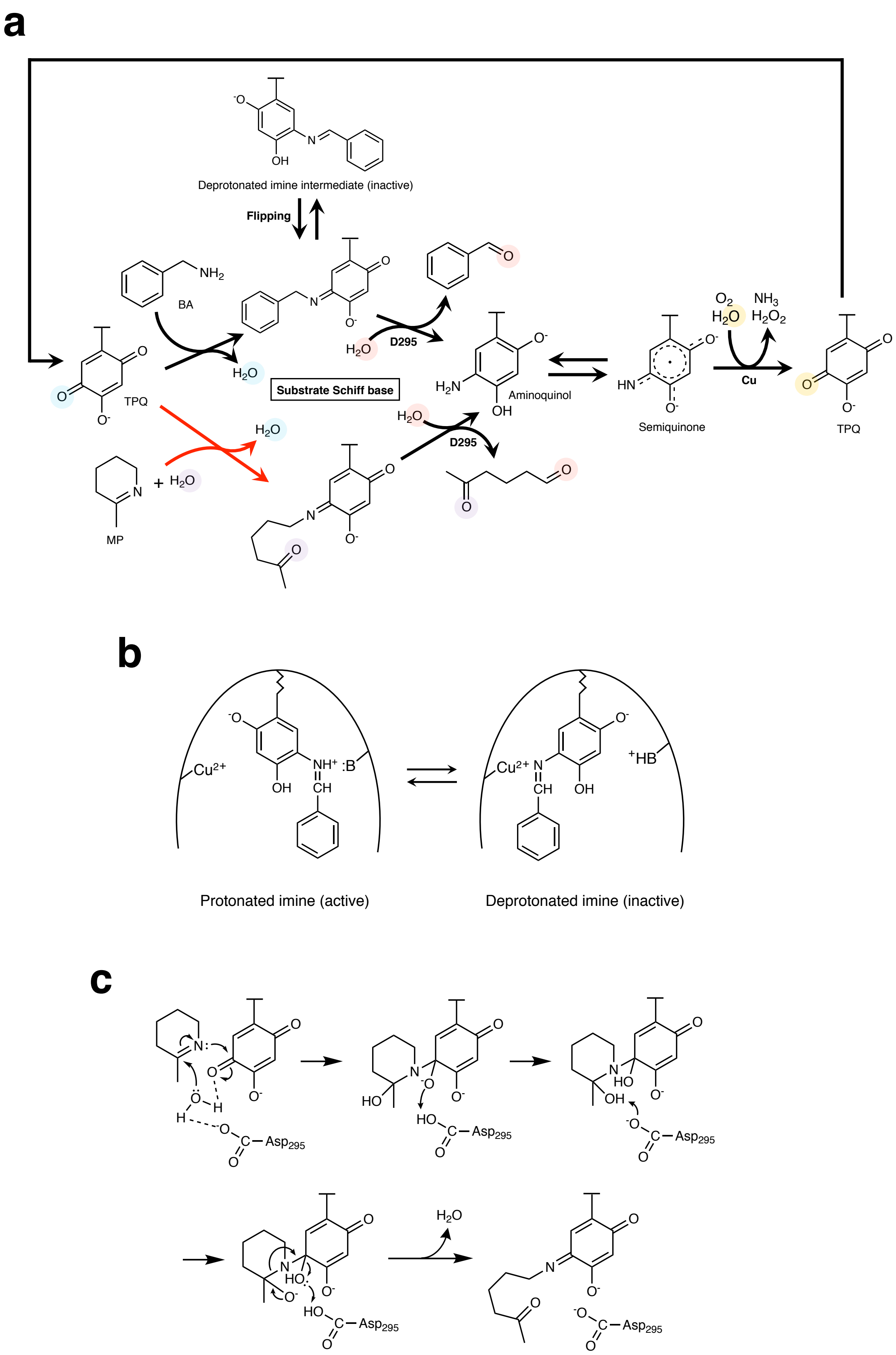
a
Deprotonated imine intermediate (inactive)
Flipping
BA
TPQ
Substrate Schiff base
D295
Aminoquinol
Semiquinone
Cu
MP
b
Protonated imine (active)
Deprotonated imine (inactive)
c
Asp295

Supplementary Figure 29. Schematic diagram of a mechanism of HarA-catalyzed reactions.

A possible reaction mechanism of HarA-catalyzed reaction is shown. (a) Schematic diagram of the HarA-catalyzed reaction when each of benzylamine (BA) and 2-methylenepiperidine (MP) was used as the substrate. The novel reaction found in this study is indicated by a red arrow. (b) Flipping of the TPQ ring in the BA adduct of HarA, based on Nakamura *et al*[9]. (c) A possible reaction mechanism of the formation of substrate Schiff base when MP was used as the substrate.

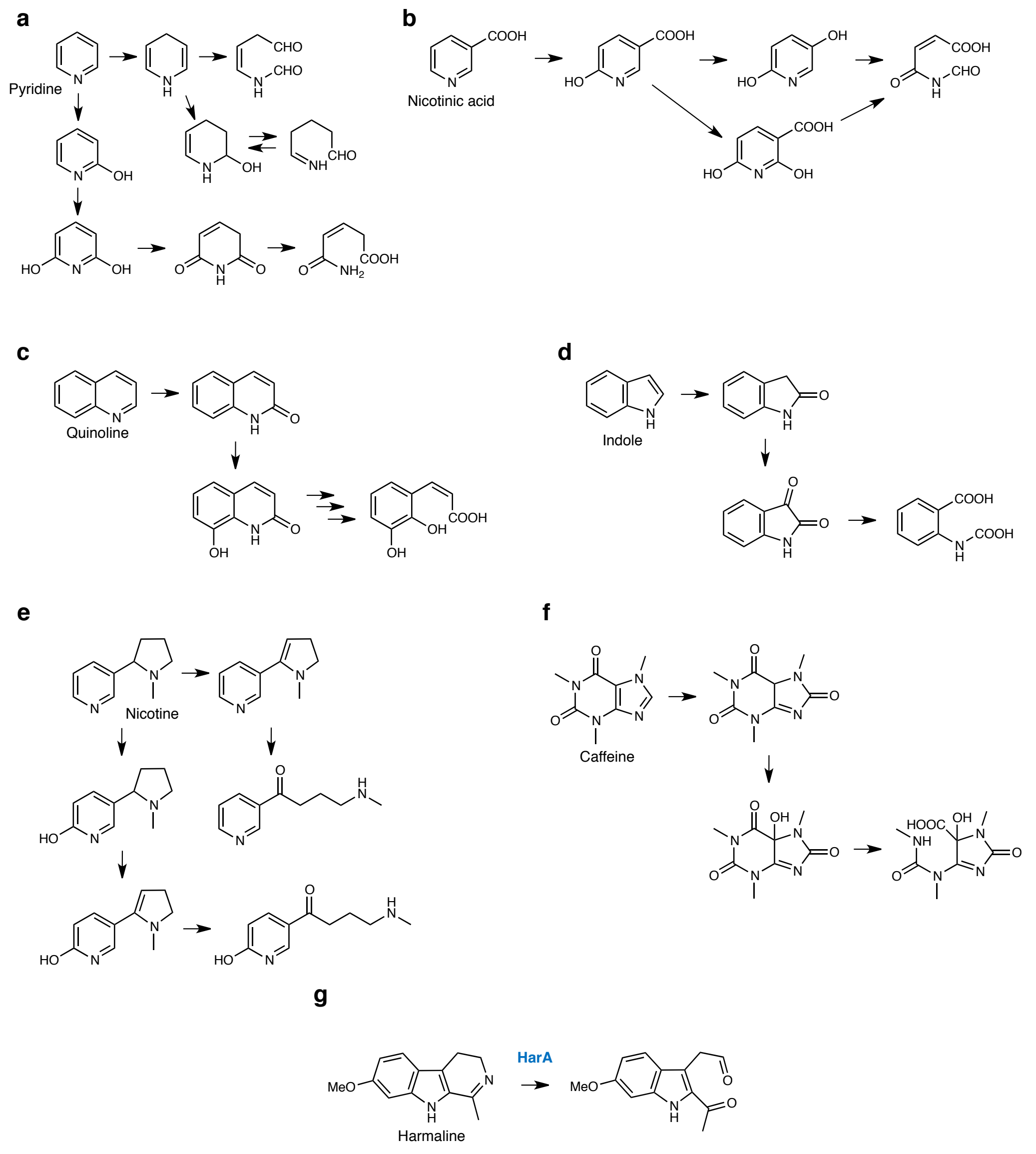

Supplementary Figure 30. Comparison of *N*-heterocyclic alkaloid-degrading pathways.

The rings of *N*-heterocyclic alkaloids are cleaved through various pathways. Pyridine (a, *Corynebacterium* sp., etc.), nicotinic acid (b, *Pseudomonas* sp., etc.), quinoline (c, *Pseudomonas fluorescens*, etc.), indole (d, *Desulfobacterium indolicum*, etc.), nicotine (e, *Pseudomonas* species and *Arthrobacter nicotinovorans*), caffeine (f, *Pseudomonas* sp. CBB1), and harmaline (g, strain C-4A, this study).

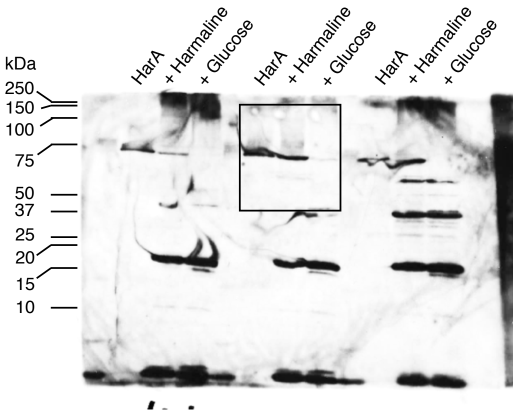

Figure 31. An uncropped blot for Figure 1e.

Cropped area is indicated by the square.

Supplementary Tables

Supplementary Table 1. Purification of HarA from strain C-4A.

Step	Protein (mg)	Total activity (nmol min^{-1})	Specific activity (nmol min^{-1} mg^{-1})	Yield (%)
Cell-free extract	1220	92.1	0.0755	100
$(NH_4)_2SO_4$ (35-80%)	403	54.8	0.136	59.5
Hiprep Butyl FF 16/10	20.5	8.59	0.419	9.33
Resource Q	1.05	2.26	2.15	2.45
BioAssist Q	0.00933	0.00764	0.821	0.00829

Supplementary Table 2. NMR spectra data for DNPH-adduct of 2-AIMA.

No.	^{1}H (ppm)	Multiplicity	*J* (Hz)	^{13}C (ppm)
1				
2				131.96
3				116.69
4				122.31
5	7.660	d (1H)	8.82	122.24
6	6.779	dd (1H)	2.28, 8.82	112.37
7				159.21
8	6.874	d (1H)	2.16	94.26
9				137.95
10	4.153	d (2H)	5.64	29.05
11	8.117	t (1H)	5.61	152.00
1’				145.47
2’	7.844	d (1H)	9.66	116.65
3’	8.813	d (1H)	2.70	123.59
4’				136.86
5’	8.330	dd (1H)	2.55, 7.02	129.39
6’				130.21
$-CH_3$	2.609	s (3H)		28.85
$-OCH_3$	3.803	s (3H)		55.65
C=O				190.47

Supplementary Table 3. NMR spectra data for the alcohol derivative of 2-AIMA.

No.	^{1}H (ppm)	Multiplicity	J (Hz)	^{13}C (ppm)
1				
2				132.09
3				120.68
4				122.9
5	7.570	d (1H)	8.82	122.37
6	6.720	dd (1H)	2.28, 8.82	111.69
7				159.06
8	6.828	d (1H)	2.16	94.34
9				137.90
10	3.188	t (2H)	5.64	29.35
11	3.594	dd (2H)	5.61	61.19
$-CH_3$	2.559	s (3H)		28.85
$-OCH_3$	3.791	s (3H)		55.65
-OH	4.743	t (1H)	4.71	

Supplementary Table 4. Bacterial strains and plasmids used in this study.

Strains	Relevant characteristics
Strain C-4A	Wild-type harmaline-metabolizing strain
E. coli DH10B	Cloning host; F^-, *mcr*A, Δ(*mrr-hsdRMS-mcrBC)*, φ80Δ*lacZ*, ΔM15, Δ*lacX74*, *deoR*, *recA1*, *araD139*, Δ(*ara leu*)7697, *galU*, *galK*, λ^-, *rpsL*, *end*A1, *nupG*
E. coli BL21 CodonPlus® (DE3) RIL	Strain for recombinant protein expression; F^-, *ompT*, *hsdS* (r_B-, m_B-), *dcm*+, Tet^r, *gal*, λ(DE3), *endA*, Hte [*argU*, *ileY*, *leuW*, CamR]

Plasmids	Relevant characteristics
pETDuet-1	T7 RNA polymerase-dependent recombinant protein expression vector, AmpR.
pETDuet-1-*harA*	The *harA* fragment (1,950 bp) was inserted into the *Nco*I and *Bam*HI sites of pETDuet-1.
pET24a(+)	T7 RNA polymerase-dependent recombinant protein expression vector, KanR.
pET24a(+)-*tynA*	The *tynA* fragment (2,274 bp) was inserted into the *Nde*I and *Bam*HI sites of pET24a(+).

Supplementary Table 5. Primers used in this study.

harA Forward (NcoI)	TAAGAAGGAGATATACCATGCACAGCCCGCTATTCGACTCCCTGACC
harA Reverse (BamHI)	GCCGAGCTCGAATTCCTAGTGCACGCAGCTGCATTGCCCG
Y281A Forward	GCCGGTATCCCGGAAGCCGCCCACTACT
Y281A Reverse	CGGCACTATCATCTCGGCGAGTGAG
D295A Forward	GCCGCCGGTGAGTACGGATTGGGCAAAA
D295A Reverse	GAATGCGCTGCGGAAGTAGTGGGCG
D295E Forward	GAGGCCGGTGAGTACGGATTGGGCAAAA
N378A Forward	GCCTACCACTACGGCTTCTACTGGACT
N378A Reverse	GCCAATTGTTGCGATAAAGCTTACG
Y379F Forward	TTCCACTACGGCTTCTACTGGAACTTCT
Y379F Reverse	GTTGCAATTGTTGCGATAAAGCTT
H380A Forward	GCCTACGGCTTCTACTGGAACTTCTTCC
H380A Reverse	GTAGTTGCCAATTGTTGCGATAAAG
tynA Forward (NdeI)	AAGGAGATATACATATGGGAAGCCCCTCTCTGTATTCTGCCC
tynA Reverse (BamHI)	GCTCGAATTCGGATCCACTTATCTTTCTTCAGCGCCCCTAGCGTTGG

Supplementary Methods

The nucleotide sequence of *harA*.

1 ATGCACAGCCCGCTATTCGACTCCCTGACCGCAGACGAGATCACCATGGTCTCGAC
57 CCTCATCAAGCAGAAAGGCATTGGAGGTGAACGTCCGGGCTTCGGCTCGGTCTTCA
113 CACACGAGCCAGACAAAGCCAGGCTGCGGCGCGGTGACAAAGTCACGCGCCAAGCC
169 CGCGCCCTCGTCCTGGACCGCAACTCGGCGGCCACTTTCGACGTCATCGTTGACCT
225 CGAAGCTGAAGCCATTGCCTCAGTGGTCCAACTCACCGACGGCGGCGCACAAATGC
281 TCGCCGAGGAGATCGAACTGGCTGACCAAATTGCCAAGGCCTCCGCGGAGTACATC
337 GAGGCGCTTTCCAAACGAGGTATCACAGACCTCAGCCTCGTCCAGCTGGACCCGTT
393 CGGCGTCGGCAACCGTGGGGACATCGACGTCGAGGGCCGCCGCCTCTGGGCCTGCG
449 TCTCCTACTTCCGCCATTTCGAGGACGACAACGCCTACGCCCACATCATTGAGGGC
505 GTTATCGCGATCGTTGACACGGTCAGGTGCGAGGTCTATGCCATCGAAGACACCGG
561 AGTCAAACCGATGCCGATGACGTGCAACAACTACACCGCAGACGCCAATGGGCCTA
617 TGCGCGAGGACGTCAAGCGACTTGACATCCTCCAAGTCGACGGACCGGGCTTCACC
673 CTCGACGGGCCACAGATGAAGTGGCAGAAATGGCGTTTCCGGATAAACATGCACCC
729 CATCGACGGTTTGGTGCTCTCCGGCATTGAGTACGACGATAGCAAGGCGGGGCAGC
785 CCGAGAACTACCGCTCAGTGATGCACCGTGCCTCACTCGCCGAGATGATAGTGCCG
841 TACGGTATCCCGGAAGCCGCCCACTACTTCCGCAGCGCATTCGACGCCGGTGAGTA
897 CGGATTGGGCAAAATGGCGAACTCTCTCGTGCTCGGCTGTGACTGCCTCGGCGACA
953 TCACGTACCTGGACGCAGTCATGGCTGATGACGACGGCAACCCGGCGCTGATCAAG
1009 AATGCCATCTGCATTCACGAGGAAGACGCCGGCATCCTGTGGAAACACACCGACTG
1065 GGCGACCGGGAAGGTCGATGTACGCCGCTCCCGCAAGCTCGTCGTAAGCTTTATCG
1121 CAACAATTGGCAACTACCACTACGGCTTCTACTGGAACTTCTTCCAGGACGGTCAT
1177 ATAGAGGTCGACACCAAGCTGATGGGAGTGGTGCAAACGATTTCGTATGAGGATGG
1233 ACAAGTGCCGCCAAACGCCAGCCCTATCGGGGAAAACCTCGCGGCGACGTGGCACC
1289 AGCACCTGTTCAACTTCCGTCTGGACATGGAGGTCGACGGCTGGCAAAACTCCGTC
1345 TATCAGAACGACGTCGACGGCGAGCCCGTCGGGCCTGCCAACCCATATGGCAACGC
1401 CATCCGGGCCACCAAGACTCTGATCGCCCGCGAGCAGGACGGCGACGGCTTGGCCA
1457 ACCCACAGACTGCACGCTCGTGGACCGTGGCCAACCCCAATAAGCACAACGCATGG
1513 GGCGTCCCGGTCGGCTACAAGCTCCTCCCGGGCTGGGCTTCGGACACGCTGATTGC
1569 TCAGGAGCCGTCGCTGATGGTCAAGCGCGCCGGGTTCGCCACTAAGAACATGTGGG
1625 TGACACCGTACGCCCCCGATGAGATGCACTCCGCGGGGGACCATCCTAACCAGGAC
1681 CGCGCAGGAGCAGGTCTCCCGGCCTGGACTGCCGCGAACCGACCGGTGGAGAACAC
1737 CGACGTCGTGCTTTGGCACACCGTAGGAGTAACACACATTCCACGCTCTGAAGACT
1793 GGCCCGTCATGCCGAACGAGACCGCGAGCTTCATGCTCGTGCCCAACAACTTCTTC
1849 GATAAAAACCCCGCACTGGACGTGCCGGACCTCAGCATGGACCGCTGCGCTCCGGG
1905 TGGCTGCTCGCACTGCCCTCCCGGGCAATGCAGCTGCGTGCACTAG

The amino acid sequence of HarA.

```
1   MHSPLFDSLTADEITMVSTLIKQKGIGGERPGFGSVFTHEPDKARLRRGDKVTRQARALV
61  LDRNSAATFDVIVDLEAEAIASVVQLTDGGAQMLAEEIELADQIAKASAEYIEALSKRGI
121 TDLSLVQLDPFGVGNRGDIDVEGRRLWACVSYFRHFEDDNAYAHIIEGVIAIVDTVRCEV
181 YAIEDTGVKPMPMTCNNYTADANGPMREDVKRLDILQVDGPGFTLDGPQMKWQKWRFRIN
241 MHPIDGLVLSGIEYDDSKAGQPENYRSVMHRASLAEMIVPYGIPEAAHYFRSAFDAGEYG
301 LGKMANSLVLGCDCLGDITYLDAVMADDDGNPALIKNAICIHEEDAGILWKHTDWATGKV
361 DVRRSRKLVVSFIATIGNYHYGFYWNFFQDGHIEVDTKLMGVVQTISYEDGQVPPNASPI
421 GENLAATWHQHLFNFRLDMEVDGWQNSVYQNDVDGEPVGPANPYGNAIRATKTLIAREQD
481 GDGLANPQTARSWTVANPNKHNAWGVPVGYKLLPGWASDTLIAQEPSLMVKRAGFATKNM
541 WVTPYAPDEMHSAGDHPNQDRAGAGLPAWTAANRPVENTDVVLWHTVGVTHIPRSEDWPV
601 MPNETASFMLVPNNFFDKNPALDVPDLSMDRCAPGGCSHCPPGQCSCVH
```

Supplementary Notes

Supplementary Note 1

The HarA-coding gene (named *harA*) was amplified from the genome of strain C-4A. An expression plasmid containing *harA* was then constructed and introduced into *E. coli*. We then investigated the effects of the addition of various metal ions to the medium on the HarA activity of HarA-expressing *E. coli* and strain C-4A. When transformed *E. coli* and strain C-4A were grown in a medium containing each of the supplemented various metal ions, only Cu^{2+} gave obvious increases in the HarA activity in their cell-free extracts (Supplementary Fig. 2). We thus used the Cu^{2+}-supplemented medium for the preparation of recombinant HarA for further analysis. HarA was heterologously expressed in *E. coli* and purified to homogeneity (Supplementary Fig. 3). The specific activity of the purified recombinant HarA was 1.67 nmol min^{-1} mg^{-1}. The copper content of HarA was measured by atomic absorption spectroscopy and calculated to be 0.303 per HarA monomer. Furthermore, HarA was found to be inhibited by a strong chelator, dimethyldithiocarbamate (Supplementary Fig. 4).

We next examined the effects of temperature and pH on the HarA activity. The optimal reaction temperature and pH were 35°C and 7.5, respectively (Supplementary Fig. 5a, b). HarA was most stable at 20°C and pH 9.5 (Supplementary Fig. 5c, d).

Supplementary Note 2

The structure of the reaction product of HarA-catalyzed harmaline degradation was determined as follows. High-resolution mass spectrometry (HRMS) analysis in the positive ion mode revealed the ion of the reaction product at *m/z* 232.0975 $[M+H]^+$, which was in agreement with the calculated mass of $C_{13}H_{14}NO_3$, 232.0974. On comparison of this molecular formula with that of harmaline ($C_{13}H_{14}N_2O$), we speculated that the reaction product had aldehyde and ketone groups, both of which were formed through a HarA-catalyzed ring-opening reaction (Fig. 2a).

In order to conduct nuclear magnetic resonance (NMR) analysis for structure determination of the reaction product, we prepared it using recombinant HarA. However, the reaction product underwent denaturation on evaporation of the solvent used

for purification, likely due to the electrophilic reactivity of the aldehyde group of the reaction product. The reaction product was thus prepared again through the modified procedure (described under Derivatization and Structure Determination of the Reaction Product), including initial 2,4-dinitrophenylhydrazine (DNPH) derivatization of the aldehyde group (Supplementary Fig. 6). The structure of the DNPH-derivatized reaction product was then determined by NMR analysis (Fig. 2b, Supplementary Figs. 7-11, and Supplementary Table 2). This hydrazone-containing structure demonstrated that the underivatized reaction product, 2-acetyl-1*H*-indol-6-methoxy-3-acetaldehyde (2-AIMA), has an aldehyde group at C-11, which reacts with DNPH (Fig. 2a).

Supplementary Note 3

We examined whether 2-AIMA was further metabolized by strain C-4A. 2-AIMA was converted into a new product, which exhibited *m/z* 232 $[M-H]^-$ on LC/MS analysis, by cell free extracts of strain C-4A on the addition of NADPH (Supplementary Fig. 13). The determined structure of the new product (Supplementary Figs. 14-18) indicated that 2-AIMA was metabolized to its alcohol derivative by an NADPH-dependent enzyme in strain C-4A.

Supplementary Note 4

We investigated a mechanism of oxygen atom incorporation into 2-AIMA. We expected that if the two additional oxygen atoms of 2-AIMA had been derived from H_2O, we would have found a double ^{18}O-labelled product in the $H_2{}^{18}O$-containing reaction mixture. In the reaction mixture that contained 90% of $H_2{}^{18}O$, however, a *m/z* 230 $[M-H^-]^-$ product (not labelled) and a *m/z* 232 $[M-H^-]^-$ product (single ^{18}O-labelled) were detected with the standard method of LC/MS analysis (Supplementary Fig. 20a-d). For single ^{18}O-labelled 2-AIMA, ^{18}O was included in the ketone group of the product (Supplementary Fig. 21). Using $^{18}O_2$ and $H_2C^{18}O_3$, we confirmed that an oxygen atom was not incorporated into the reaction product from atmospheric O_2 or CO_2. In addition, when 2-AIMA which was enzymatically synthesized in $H_2{}^{16}O$ was incubated in $H_2{}^{18}O$ without the enzyme, one of ^{16}O of 2-AIMA was exchanged with ^{18}O (Supplementary Fig. 21e,f). These findings demonstrated that the oxygen atom of the aldehyde group of 2-AIMA had been rapidly exchanged with that of H_2O.

We purified 2-AIMA from the above reaction mixture by ethyl acetate extraction,

and then analyzed the resulting solution by LC/MS without a column and an aqueous carrier. As a result, a *m/z* 234 $[M\text{-}H^-]^-$ product (double ^{18}O-labelled) was successfully detected with the above modified method of LC/MS analysis (Fig. 2h).

Supplementary Note 5

We investigated the stability of cyclic imine within harmaline (Supplementary Figure 28). LC/MS analyses including derivatization of carbonyl and amine groups showed that clear signal of spontaneously hydrolyzed harmaline was not detected in water-based solution. On the other hand, hydrolytic products of linear imine (benzylidenemethylamine) was easily detected.

Supplementary Discussion

Comparison of metabolic pathways of N-heterocyclic alkaloids

In so far known degradation pathways for *N*-heterocyclic alkaloids, two or more enzymes are usually involved in the ring cleavage (Supplementary Fig. 30). The ring moiety of an *N*-heteroaromatic alkaloid is very often subjected to initial hydroxylation by a hydroxylase or monooxygenase (Supplementary Fig. 30a-d)[1]. In general, the resultant hydroxylated heteroaromatic ring is cleaved via the following dioxygenolytic reaction[1]. There are also some pathways involving several hydroxylation and hydrogenation steps prior to the ring cleavage[1]. On the other hand, non-aromatic *N*-heterocyclic rings are subjected to hydrolysis after some modifications (Supplementary Fig. 30e, f)[2,3]. In this study, we discovered that cyclic imine within harmaline was degraded by the single enzyme, HarA, from a harmaline-metabolizing microorganism (Supplementary Fig. 30g). This reaction is very different from the canonical multi-step ring-opening of an *N*-heterocyclic alkaloid. Growth of strain C-4A in the minimum medium containing harmaline as the sole nitrogen source suggests that the degradation of the cyclic imine within harmaline by HarA enables strain C-4A to efficiently acquire nitrogen from harmaline. Although the aldehyde product, 2-AIMA, would be highly reactive and toxic for most enzyme catalyst, we found that 2-AIMA was metabolized to the corresponding stable alcohol metabolite by a NADPH-dependent enzyme in C-4A (Supplementary Figs. 13-18).

Investigation of bacterial metabolism for heterocyclic alkaloids

Although numerous chemical reactions have been found so far, intriguing enzymatic reactions remain unidentified, especially in underground environments. A number of microorganisms may use these overlooked reactions to adapt to various conditions. To elucidate how such microorganisms make use of unknown enzymatic reactions will lead to the discovery of further potential of biocatalysts for their survival and growth in the presence of antibiotics. Although harmaline exhibits antibacterial activity against broad species of microorganisms[4-6], strain C-4A was able to grow well in the presence of harmaline, inducing the expression of HarA (Fig. 1d, e). Some ring-opening enzymes

(e.g., β-lactamase and epoxidase), which act on heterocyclic substrates, are observed in the microbial inactivation of clinically important antibiotics[7-9]. Exploring microbial metabolism of heterocyclic compounds could result in not only the discovery of new enzyme functions, but also provide tools for predicting potential antibiotic resistances to novel antibiotic containing heterocyclic structures.

Supplementary References

1. Fetzner, S. Bacterial degradation of pyridine, indole, quinoline, and their derivatives under diferrent redox conditions. *Appl. Microbiol. Biotechnol.* **49**, 237-250 (1998).
2. Brandsch, R. Microbiology and biochemistry of nicotine degradation. *Appl. Microbiol. Biotechnol.* **69**, 493-498 (2006).
3. Summers, R. M., Mohanty, S. K., Gopishetty, S. & Subramanian, M. Genetic characterization of caffeine degradation by bacteria and its potential applications. *Microb. Biotechnol.* **8**, 369-378 (2015).
4. Arshad, N., Zitterl-Eglseer, K., Hasnain, S. & Hess, M. Effect of *Pegenum harmala* or its β-carboline alkaloids on certain antibiotic resistant strains of bacteria and protozoa from poultry. *Phytother. Res.* **22**, 1533-1538 (2008).
5. Ahmad, A. *et al.* Study of the in vitro antimicrobial activity of harmine, harmaline and their derivatives. *J. Ethnopharmacol.* **35**, 289-294 (1992).
6. Xing, M. *et al.* Antimicrobial efficacy of the alkaloid harmaline alone and in combination with chlorhexidine digluconate against clinical isolates of *Staphylococcus aureus* grown in planktonic and biofilm cultures. *Lett. Appl. Microbiol.* **54**, 475-482 (2012).
7. Wright, G. D. Bacterial resistance to antibiotics: enzymatic degradation and modification. *Adv. Drug Deliv. Rev.* **57**, 1451-1470 (2005).
8. Martínez, J. L. Antibiotics and antibiotic resistance genes in natural environments. *Science* **321**, 365-367 (2008).
9. Blair, J. M., Webber, M. A., Baylay, A. J., Ogbolu, D. O. & Piddock, L. J. V. Molecular mechanisms of antibiotic resistance. *Nat. Rev. Microbiol.* **13**, 42-51 (2015).
